# Top-Down Proteomics of Medicinal Cannabis

**DOI:** 10.3390/proteomes7040033

**Published:** 2019-09-24

**Authors:** Delphine Vincent, Steve Binos, Simone Rochfort, German Spangenberg

**Affiliations:** 1 Agriculture Victoria Research, AgriBio, Centre for AgriBioscience, Bundoora, Victoria 3083, Australia; simone.rochfort@agriculture.vic.gov.au (S.R.); german.spangenberg@agriculture.vic.gov.au (G.S.); 2 Thermo Fisher Scientific, Bio21 Institute, The University of Melbourne, 30 Flemington Rd, Parkville, Victoria 3052, Australia; steve.binos@thermofisher.com

**Keywords:** infusion, LC-MS, LC-MS/MS, SID, CID, HCD, ETD, PTMs, apical bud, cannabis intact proteins, protein standards, proteoforms, top-down sequencing, Prosight Lite, Mascot

## Abstract

The revised legislation on medicinal cannabis has triggered a surge of research studies in this space. Yet, cannabis proteomics is lagging. In a previous study, we optimised the protein extraction of mature buds for bottom-up proteomics. In this follow-up study, we developed a top-down mass spectrometry (MS) proteomics strategy to identify intact denatured protein from cannabis apical buds. After testing different source-induced dissociation (SID), collision-induced dissociation (CID), higher-energy collisional dissociation (HCD), and electron transfer dissociation (ETD) parameters on infused known protein standards, we devised three LC-MS/MS methods for top-down sequencing of cannabis proteins. Different MS/MS modes produced distinct spectra, albeit greatly overlapping between SID, CID, and HCD. The number of fragments increased with the energy applied; however, this did not necessarily translate into greater sequence coverage. Some precursors were more amenable to fragmentation than others. Sequence coverage decreased as the mass of the protein increased. Combining all MS/MS data maximised amino acid (AA) sequence coverage, achieving 73% for myoglobin. In this experiment, most cannabis proteins were smaller than 30 kD. A total of 46 cannabis proteins were identified with 136 proteoforms bearing different post-translational modifications (PTMs), including the excision of N-terminal M, the N-terminal acetylation, methylation, and acetylation of K resides, and phosphorylation. Most identified proteins are involved in photosynthesis, translation, and ATP production. Only one protein belongs to the phytocannabinoid biosynthesis, olivetolic acid cyclase.

## 1. Introduction

The state of Victoria in Australia was the first jurisdiction to legalise access to medicinal cannabis under the Medicinal Cannabis Act in 2016 (www2.health.vic.gov.au). In this context, we have controlled access to medicinal cannabis material grown to full maturity in the state-of-the-art Victorian government medicinal cannabis cultivation facility. Utilizing this asset, we developed analytical methods for bottom-up proteomics of mature buds [1] and cannabinoid quantitation from cannabis resin [2]. A wealth of information pertaining to botany, phytochemistry, biology, and medicinal properties of *Cannabis sativa* has been accumulated over the years since the abolition of the legal use of cannabis in 1937 [3,4]. Recent phylogenetics studies have redefined the *Cannabaceae* family to now include not only *Cannabis* and *Humulus* (hop) but also eight genera formerly assigned to the *Celtidaceae* family: *Celtis, Pteroceltis, Aphananthe, Chaetachme, Gironniera, Lozanella, Trema,* and *Parasponia* [5]. Other closely related families are *Moraceae* and *Urticaceae,* which comprise *Boehmeria nivea* (Chinese grass). A search for amino acid (AA) sequences in UniprotKB database (www.uniprot.org) using the key word “Cannabis sativa” retrieves entries from not only *C. sativa* and subspecies but also from closely related species *Humulus lupulus*, *Humulopsis scandens*, *Humulus yunnanensis*, and *Boehmeria nivea* (also called *Urtica nivea*). Another well studied area of research relates to the discovery of *C. sativa* active components, phytocannabinoids [6,7,8], and the elucidation of their biosynthetic pathway [9,10]. Owing to the change in legislation in the 1990s, an exponential surge of cannabis studies can be seen with close to 20,000 publications to date listed in Pubmed (www.ncbi.nlm.nih.gov/pubmed). The most dynamic field of cannabis research now targets medical applications. Proteomics research on *C. sativa* is greatly lagging behind, with only nine reports thus far. Five studies processed nonreproductive organs from immature cannabis plants such as roots [11], hypocotyls [12], and leaves [13] or processed seeds from hemp [14,15]. To our knowledge, only four proteomics reports have been published thus far on reproductive organs from *C. sativa*. Raharjo and colleagues in 2004 published proteomics results on flowers and glands from cannabis plants [16]. Ten years later, Happyana produced his PhD thesis on cannabis trichomes [17], and preliminary results from Jenkins and Osburn were very recently made available on the bioRxiv preprint server [18]. Early this year, we published results on shotgun proteomics [1] in which we demonstrated the superiority of protein extraction from *C. sativa* mature buds using a guanidine-hydrochloride buffer over a urea-based solution.

The term proteoforms, coined in 2016 [19], corresponds to all of the different molecular forms in which the protein product of a single gene can be found; this includes all forms of genetic variation, alternative splicing of RNA transcripts, and post-translational modifications (PTMs). Typical PTMs include phosphorylations, glycosylations, oxidations, and N-terminus acetylations [20]. Top-down proteomics describes the analysis of intact proteins, either in their native form or more often in a denatured state, which allows for a characterisation of proteoforms as comprehensive as possible [21]. Whilst gel-based proteomics such as two-dimensional electrophoresis technically fit within this definition, top-down proteomics is typically performed using gel-free- and mass spectrometry (MS)-based strategies, along with the acquisition of MS/MS spectra for sequencing purposes. A literature search within Pubmed using the key words “top-down proteomics” listed 713 publications from 2000 until 12 June 2019, including 130 reviews. The number of publications in this space is on the rise, with only a handful in the early 2000s and 82 articles in 2018. This year, already 46 papers are available in this field. Such is the interest in this emerging field that a Consortium for Top-Down Proteomics (CTDP, http://topdownproteomics.org/) was created in 2014 [22] making available relevant documentation and online resources. This consortium standardized the notation of proteoforms [23] and just released some excellent guidelines for TDP [24].

Current MS/MS fragmentation methods include collision-induced dissociation (CID), in-source CID (also known as source-induced dissociation, SID), higher-energy collisional dissociation (HCD), electron capture dissociation (ECD), electron transfer dissociation (ETD), and ultraviolet photodissociation (UVPD). Combining various fragmentation modes improves top-down sequencing outcomes, as different types of fragment ions are generated. For instance, SID, CID, and HCD create b- and y-type ions, while ETD creates c- and z-type ions. Extensive AA sequence coverage of known intact proteins was achieved through efficient multimodal gas-phase fragmentation and sophisticated computational analysis [25]. Top-down proteomic experiments are challenging for the following reasons: low abundance of many proteoforms and low signal-to-noise (S/N) ratios inherent to mass measurement of large molecules, data analysis of complex MS and MS/MS spectra, and co-elution of proteoforms during the initial online separation stage by liquid chromatography (LC) or capillary zone electrophoresis (CZE) [20]. Proteoforms whose molecular weights (MW) exceed ~50–70 kDa remain difficult to top-down sequence due to limitations inherent to MS and the need for improved separations. New generation instruments such as the 21 Tesla Fourier transform ion cyclotron resonance (21T FT-ICR) mass spectrometer, offering extended mass ranges combined with recording short time-domain transients to maximise spectral signal-to-noise ratio (S/N) of large proteoforms, could alleviate these issues [26]. Given the paucity of top-down proteomics reports on plants species, it seems the technical challenges are even greater for samples originating from plants. 

We previously published three studies on top-down proteomics of bovine milk samples using ultra-performance liquid chromatography (UPLC) online with a quadrupole time-of-flight (Q-ToF) mass spectrometer offering a 60,000 resolving power [27,28,29]. In these reports, major known milk proteins were targeted by first optimizing analytical methods on purchased standards and validating them on milk samples [28,29] and then applying the optimum method to a study aimed at exploring UHT milk (long life, ultra-heat treatment applied) properties during shelf-life storage [27]. Top-down sequencing information was obtained from CID and/or in-source CID experiments, which were the only fragmentation modes offered by this Q-ToF mass spectrometer. Data analysis proved particularly challenging, as we did not have the proprietary software from the mass spectrometer’s manufacturer required to annotate MS/MS data in an automated fashion. Ultimately, we used the Genedata Expressionist software to develop workflows, allowing us to automate batch processing and annotation of both LC-MS and LC-MS/MS data [29]. This led to the discovery of extensive proteolysis of milk major proteins—in particular, beta- and alpha-S1-caseins—during the one-year storage of UHT milk [27]. Our experience on samples sourced from animals led us to tackling a top-down proteomics study on plant tissues. This work follows on from our optimization of protein extraction of mature buds from medicinal cannabis prior to bottom-up proteomics [1]. In this top-down study, we used a hybrid mass spectrometer composed of a linear ion trap (LTQ or ITMS) and a Fourier-transform Orbitrap (FTMS), which we typically use for bottom-up experiments [1,30]. This powerful instrument offers high resolution (up to 240,000) and multiple modes of fragmentations (SID, CID, HCD, and ETD) relative to the Q-ToF system employed previously.

In a first step, we benchmarked our analytical workflow—in particular, MS and data mining—using readily available proteins. To this end, we infused known protein standards of various MWs, namely myoglobin 17 kD, alpha-casein (α-CN) 24 kD, beta-lactoglobulin (β-LG) 19 kD, and bovine serum albumin (BSA, 67 kD), and applied different MS/MS modes at increasing energy levels to assess fragmentation efficiency across different proteins and charge states of precursor ions. Our results show that different MS/MS modes produced distinct spectra, albeit greatly overlapping between SID, CID, and HCD, as they produced the same b- and y-types of ions. The number of fragment ions increased with the energy applied; however, this did not necessarily translate into greater sequence coverage. We also indicate that some precursors were more amenable to fragmentation than others, and that sequence coverage decreased as the mass of the protein increased. Combining all MS/MS data maximised AA sequence coverage, and processing it using ProSight Lite software yielded up to 73% for myoglobin, 56% for β-LG, 41% for α-S1-CN, and 6% for BSA. These initial tests helped us refine the methods to top-down sequence intact protein extracts from cannabis buds in triplicate. Following an extended 120 min separation by LC, intact denatured cannabis proteins were analysed by FTMS with the highest resolution in duplicate. They were then analysed by FTMS/MS using three methods in succession applying “low”, “medium”, and “high” energy levels for ETD, CID, and HCD modes. In this experiment, most cannabis proteins were smaller than 30 kD. Several databases were searched, more or less extensively and specifically, with more or less stringent tolerance. Out of the 11,250 MS/MS spectra generated and searched using the most specific database and highest stringency, only 258 (2%) were annotated, amounting to 46 protein accessions and detecting various PTMs (oxidation, methylation, acetylation, phosphorylations). Our results are discussed hereafter. 

## 2. Materials and Methods 

**Figure 1:**
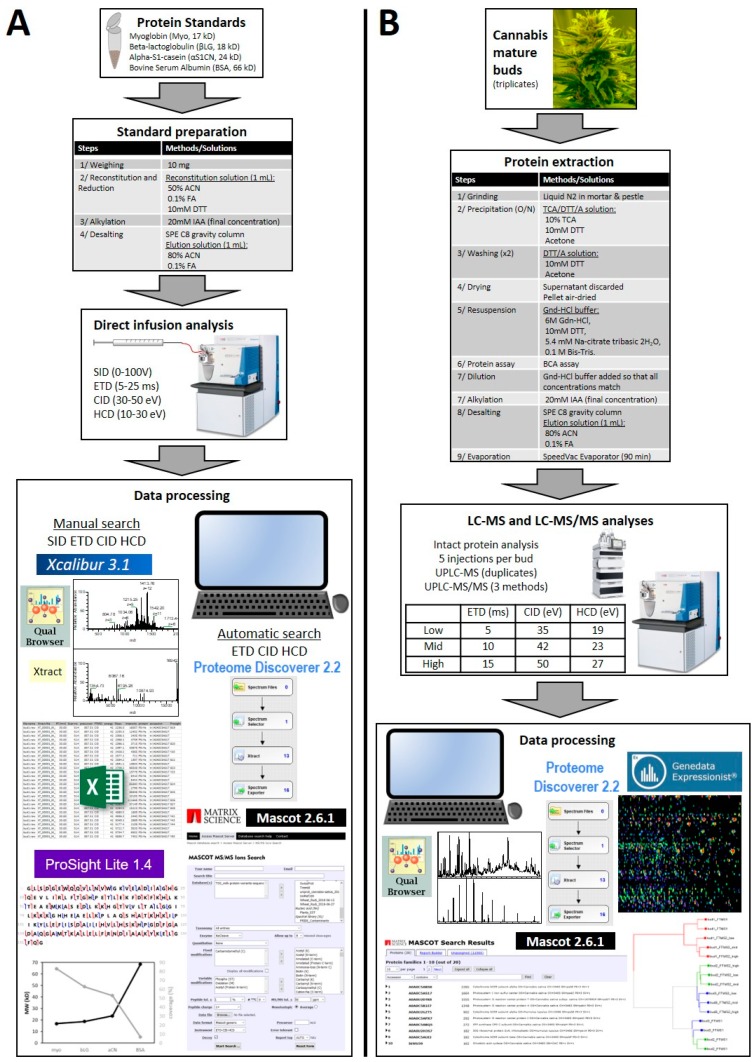
Experimental design. (**A**) Processing of known protein standards; (**B**) processing of cannabis bud samples.

The experimental design is schematised in Figure 1.

### 2.1. Protein Standards

#### 2.1.1. Standard List

The protein standards were all purchased from Sigma and included: α-casein (α-CN 23.6 kDa) from bovine milk (C6780-250MG, 70% pure), β-lactoglobulin (β-LG, 18.7 kDa) from bovine milk (L3908-250MG, 90% pure), albumin from bovine serum (BSA, 66.5 kDa, A7906-10G, 98% pure), and myoglobin from horse skeletal muscle (Myo, 16.9 kDa, M0630-250MG, 95–100% pure and salt-free) [28,29].

#### 2.1.2. Standard Preparation

These lyophilized protein standards were fully solubilised at a 10 mg/mL concentration in 50% acetonitrile (ACN)/0.1% formic acid (FA)/10 mM dithiothreitol (DTT). Standards were dissolved by vortexing for 1 min and sonication for 10 min followed by another 1 min vortexing. Iodoacetamide (IAA) was added to reach a final concentration of 20 mM, vortexed for 1 min, and left to incubate for 30 min at room temperature in the dark. Apart from BSA and β-lactoglobulin, none of the standards needed reduction and alkylation steps, as they had no disulfide bridges; yet, these steps were still performed to emulate plant sample processing.

Standard solutions were then desalted using solid phase extraction (SPE) cartridges (Sep-Pak C18 1cc Vac Cartridge, 50 mg sorbent, 55–105 μm particle size, 1 mL, Waters, Milford, MA, USA) by gravity as described in [30]. Bound intact proteins were desalted using 1 mL of 0.1% FA solution and eluted into a 2 mL microtube using 1 mL of 80% ACN/0.1% FA solution.

### 2.2. Plant Materials

#### 2.2.1. Cannabis Sampling and Grinding

Fresh plant material was obtained from the Victorian Government Medicinal Cannabis Cultivation Facility. The top three centimeters of the mature apical bud were excised using secateurs, placed into a labelled paper bag, snap frozen in liquid nitrogen, and stored at −80 °C until grinding. Samples were collected in triplicate. 

Frozen buds were ground in liquid nitrogen using a mortar and pestle. The ground frozen powder was transferred into a 15 mL tube and stored at stored at −80 °C until protein extraction.

#### 2.2.2. Cannabis Protein Extraction

Protein extraction for cannabis mature apical buds was previously optimized [1], with the method referred to as “Extraction 4” yielding the best results. This method was upscaled as detailed below.

One 500 mg scoop of ground frozen powder was transferred into a 15 mL tube kept on ice prefilled with 12 mL ice-cold 10% trichloroacetic acid (TCA)/10mM dithiothreitol (DTT)/acetone (*w*/*w*/*v*). The tubes were vortexed for 1 min and left at −20 °C overnight. The next day, tubes were centrifuged for 30 min at 4 °C and at maximum speed (5000 rpm) using a swing rotor centrifuge (Sigma, (Australia) 4–16 k). The supernatant was removed, and the pellet was resuspended in 12 mL ice-cold 10 mM DTT/acetone (*w*/*v*) by vortexing for 1 min. Tubes were left at −20 °C for 2 h. The tubes were centrifuged as specified before, and the supernatant was removed. This washing step of the pellet was repeated once more. The pellets were dried for 30 min under a fume hood. The dry pellet was resuspended in 2 mL of guanidine-HCl buffer (6 M guanidine-HCl, 10 mM DTT, 5.37 mM sodium citrate tribasic dihydrate and 0.1 M Bis-Tris).

#### 2.2.3. Protein Assay and Cannabis Protein Alkylation 

Protein extracts from apical buds were diluted ten times into guanidine-HCl buffer. The protein concentrations were measured in triplicate using the Microplate BCA protein assay kit (Thermo Scientific, MA, USA) following the manufacturer’s instructions. Bovine serum albumin (BSA) from the kit was used as a standard as per instructions. Protein extract concentrations ranged from 2.84 to 3.72 mg of proteins per mL of extract.

Following protein assay, the concentrations of the DTT-reduced protein samples were adjusted to that of the least concentrated (2.84 mg/mL) by adding the appropriate volume of guanidine-HCl buffer. The protein extracts were then alkylated by adding a volume of 1 M iodoacetamide (IAA)/water (*w*/*v*) solution to reach a 20 mM final IAA concentration. The tubes were vortexed for 1 min and left to incubate at room temperature in the dark for 60 min.

#### 2.2.4. Cannabis Protein Desalting and Evaporation

A volume of 0.5 mL of alkylated protein extract (1.42 mg proteins) was then desalted as described above in Section 2.1.2.

The 1 mL eluates were then evaporated using a SpeedVac concentrator (Savant SPD2010, Thermo Scientific, Waltham, MA, USA) for 90 min until the volume reached 0.2 mL. The evaporated samples were transferred into a 100 μL glass insert placed into a glass vial. The vials were positioned into the autosampler at 4 °C for immediate analyses by UPLC-MS.

### 2.3. Mass Spectrometry Analyses

MS analyses were performed on an Orbitrap Elite hybrid ion trap-Orbitrap mass spectrometer (Thermo Scientific, MA, USA) composed of a linear ion trap quadrupole (ITMS) mass spectrometer hosting the source and a Fourier transform mass spectrometer (FTMS) with a resolution of 240,000 at 400 *m/z*. Both ITMS and FTMS were calibrated in positive mode, and the ETD was tuned prior to all MS and MS/MS experiments. All MS and MS/MS files [RAW, mzXML, Mascot Generic Format (MGF)] and FASTA files from known protein standards and cannabis samples are available from the stable public repository MassIVE at the following URL: http://massive.ucsd.edu/ProteoSAFe/datasets.jsp with the accession number MSV000083970.

#### 2.3.1. Infusion of Protein Standards and Analyses by Mass Spectrometry

Protein standard solutions were individually infused using a 0.5 mL Gastight #1750 syringe (Hamilton Co.) at a 20–30 µL/min flow rate using the built-in syringe pump of the LTQ mass spectrometer to achieve at least 1e6 ion signal intensity. Protein standard solutions were first pushed through 30 cm of red PEEK tubing (0.005 in ID) and then through a metal union and a PEEK VIPER tube (6041–5616, 130 µm × 150 mm, Thermo Fischer Scientific) to the heated electrospray ionisation (HESI) source where proteins were ionized through a HESI needle insert 0.32 gauge (Thermo Fisher Scientific 70005-60155). 

The source parameters were: capillary temperature 300 °C, source heater temperature 250 °C, sheath gas flow 30, auxillary gas flow 10, sweep gas flow 2, FTMS injection waveforms on, FTMS full AGC target 1e6, FTMS MSn AGC target 1e6, positive polarity, source voltage 4 kV, source current 100 µA, S-lens RF level 70%, reagent ion source CI pressure 10, reagent vial ion time 200 ms, reagent vial AGC target 5e5, supplemental activation energy 15V, FTMS full micro scans 16, FTMS full max ion time 100 ms, FTMS MSn micro scans 8, and FTMS MSn max ion time 1000 ms. SID was set at 15 V, and FT Penning gauge pressure difference was set at 0.01 E-10 Torr to improve signal intensity. The scanning mass windows were 600–2000 *m/z* for FTMS1 and 300–2000 *m/z* for FTMS2.

Various fragmentation parameters were tested on individual protein standards, including in-source fragmentation (SID) potentials from 0 to 100 V (maximum potential). Collision-induced dissociation (CID) normalized collision energy (NCE) varied from 30 to 50 eV with constant activation Q of 0.400 and an activation time of 100 ms. High energy CID (HCD) NCE varied from 10 to 30 eV with constant activation time of 0.1 ms. Electron transfer dissociation (ETD) activation times varied from 5 to 25 ms with constant activation Q of 0.250. Data files were acquired on the fly using the Acquire Data function of Tune Plus software 2.7 (Thermo Fisher Scientific) for up to 3 min at a time.

#### 2.3.2. Separation of Cannabis Intact Proteins by Ultra-Performance Liquid Chromatography (UPLC)

Intact proteins from cannabis mature buds were chromatographically separated using a UPLC 1290 Infinity Binary LC system (Agilent, Santa Clara, CA, USA) and a bioZen XB-C4 column (3.6 µm, 200 Å, 150 × 2.1 mm, Phenomenex, Torrance, CA, USA) kept at 90 °C. Flow rate was 0.2 mL/min, and total duration was 120 min. Mobile phase A contained 0.1% FA in water, and mobile phase B contained 0.1% FA in acetonitrile. 

Chromatographic separation was optimized (data not shown), and optimum UPLC gradient for cannabis proteins was as follows: starting conditions 3% B, ramping to 15% B in 2 min, ramping to 40% B in 89 min, ramping to 50% B in 5 min, ramping to 99% B in 5 min and held at 99% B for 10 min, lowering to 3% B in 1.1 min, equilibration at 3% B for 7.9 min. A 20 µL injection volume was applied to each protein extract. Each extract was injected five times with blanks in between. 

#### 2.3.3. Analyses of Cannabis Intact Protein Extracts using Mass Spectrometry online with UPLC

The UPLC outlet line was connected to the switching valve of the LTQ mass spectrometer. During the 119 min mass spectrometry (MS) acquisition time, the first two minutes and the last minute of the run were directed to the waste, whereas the rest of the run was directed to the source.

##### Full Scan FTMS1

Tune parameters were described above. Data were acquired in positive polarity with profile and normal scan modes at a resolution of 240,000 at 400 *m/z* along a mass window of 500–2000 *m/z*. SID was set at 15 V. Full scan files were acquired in duplicate at the first and the last injections of the 5 sample injections. The three intermediate injections were dedicated to tandem MS (see below).

##### FTMS2

Three MS/MS methods were applied in which the energy applied to each fragmentation modes varied between what we referred to as “low”, “high”, and “mid” (intermediate). SID was set to 15 V throughout. One segment was defined with four scan events. The first scan event applied full scan FTMS in profile and normal modes at a resolution of 120,000 for 400 *m/z*, scanning a mass window of 500–2000 *m/z*. The most abundant ion above an intensity threshold of 500 and *m/z* greater than 700 from the first scan was selected for subsequent fragmentation in a data-dependent manner with an isolation width of 15 and a default charge state of 10. FTMS2 spectra were acquired along a mass window of 300–2000 *m/z* at a resolution of 60,000 at 400 *m/z*. Scan events 2 to 4 are described below, as their energy levels varied. The parameters that changed are in bold.

In the “low” energy FTMS2 method, the precursor underwent ETD fragmentation during the second scan event with an activation time of 5 ms and an activation Q of 0.250; CID fragmentation in the third scan event with a NCE of 35 eV, an activation Q of 0.400, and an activation time of 100 ms; and HCD fragmentation with a NCE of 19 eV and an activation time of 0.1 ms.

In the “mid” energy FTMS2 method, the precursor underwent ETD fragmentation during the second scan event with an activation time of 10 ms and an activation Q of 0.250; CID fragmentation in the third scan event with a NCE of 42 eV, an activation Q of 0.400, and an activation time of 100 ms; and HCD fragmentation with a NCE of 23 eV and an activation time of 0.1 ms.

In the “high” energy FTMS2 method, the precursor underwent ETD fragmentation during the second scan event with an activation time of 15 ms and an activation Q of 0.250; CID fragmentation in the third scan event with a NCE of 50 eV, an activation Q of 0.400, and an activation time of 100 ms; and HCD fragmentation with a NCE of 27 eV and an activation time of 0.1 ms.

### 2.4. Data Files Analysis

#### 2.4.1. Analysis of Infusion MS/MS Spectra

##### Manual Annotations of Standards

Given the MW of myoglobin, β-lactoglobulin, α-S1-casein, and the 240,000 resolution of the instrument, the spectra of these proteins were isotopically resolved. Bovine serum albumin is too large for isotopic resolution, therefore only average mass was obtained. Isotopically resolved RAW files were opened using the Qual Browser module of Xcalibur softawe version 3.1 (Thermo Scientific) and deconvoluted using Xtract algorithm (Thermo Scientific) with the following parameters: M masses mode, 240,000 resolution at 400 *m/z* 3 S/N threshold, 44 fit factor, 25% remainder, averaging method and 40 max charges. In the deconvoluted spectra, the second scan corresponding to the monoisotopic zero-charge (deisotoped) mass spectrum was selected for export as explained in [31].

Deconvoluted exact masses were then exported to Excel 2016 (Microsoft, Redmond, WA, USA) to generate pivot tables and charts. VBA macros were used to compile lists of masses corresponding to different MS/MS modes and parameters and parent ions from the same protein. The deconvoluted, deisotoped masses were copied and pasted into ProSight Lite version 1.4 (Northwestern University, Evanston, IL, USA) with the following parameters: S-carboxamidomethyl-l-cysteine as a fixed modification, monoisotopic precursor mass type, and fragmentation tolerance of 50 ppm. The AA sequence varied according to the standards analysed; where needed, the initial methionine residue (myoglobin), the signal peptide (β-LG, α-S1-CN, BSA), and the pro-peptide (BSA) were removed. Dependent on the acquisition strategy, the fragmentation type selected was either SID, HCD, CID, or ETD. When multiple MS/MS spectra were used including ETD data, the BY and the CZ fragmentation method was selected. 

##### Automatic Annotations of Standards

Raw MS/MS files were imported into Proteome Discoverer version 2.2 (Thermo Fisher Scientific) through the Spectrum Files node, and the following parameters were used in the Spectrum Selector node: use MS1 precursor with isotope pattern, lowest charge state of 2, precursor mass ranging from 500–50,000 Da, minimum peak count of 1, MS orders 1 and 2, collision energy ranging from 0–1000, full scan type. The selected spectra were then deconvoluted through the Xtract node with the following parameters: S/N threshold of 3, 300–2000 *m/z* window, charge from 1–30 (maximum value), resolution of 60,000, and monoisotopic mass. When not specified, default parameters were used. Deconvoluted spectra (MH^+^) were then exported as a single MGF file.

The MGF file was searched in Mascot version 2.6.1 (MatrixScience, Boston, MA, USA) with top-down searches licence. An MS/MS ion search was performed with the NoCleave enzyme, carbamidomethyl (C) as fixed modification and oxidation (M), acetyl (protein N-term), and phospho (ST) as variable modifications, with monoisotopic masses, 1% precursor mass tolerance, ±50 ppm or ±2 Da fragment mass tolerance, precursor charge of +1, 9 maximum missed cleavages, and instrument type that accounted for CID, HCD, and ETD fragments (i.e., b-, c-, y-, and z-type ions) of up to 110 kDa. The no-enzyme option (“none”) was also tested but yielded fewer hits and therefore is not presented here. The first database searched was a FASTA file containing the AA sequences of all the known variants of cow’s milk’s most abundant proteins (all caseins, alpha-lactalbumin, beta-lactoglobulin, and BSA) along with horse’s myoglobin (59 sequences in total, [28]). The decoy option was selected. The second database searched was SwissProt (all 559,228 entries, version 5, last updated on 8 March 2019) using all the entries or just the “other mammalia” taxonomy.

#### 2.4.2. Analysis of LC-MS and LC-MS/MS Data from Cannabis Samples

##### Statistical Analyses of Cannabis Samples

The RAW files were loaded and processed in the Refiner modules of Genedata Expressionist ^®^ version 12.0.6 using the following steps and parameters: profile data cutoff of 10,000, R window of 3–99 min, *m/z* window of 500–1800 Da, removal of RT structures <4 scans, removal of *m/z* structures <5 points, smoothing of chromatogram using a 5 scans window and moving average estimator, spectrum smoothing using a 3 points *m/z* window, a chromatogram peak detection using a summation window of 15 scans, a minimum peak size of 1 min, a maximum merge distance of 10 ppm,, and a curvature-based algorithm with local maximum and FWHM boundary determination, isotope clustering using a peptide isotope shaping method with charges ranging from 2–25 (maximum value) and monoisotopic masses, singleton filtering, and charges and adduct grouping using a 50 ppm mass tolerance, positive charges, and dynamic adduct list containing protons, H_2_O, K-H, and Na-H. The protein groups were used for statistical analyses.

Spectral deconvolution from 3–70 kDa was performed using manual deprecated mode and harmonic suppression deconvolution method with a 0.04 Da step as well as curvature-based peak detection, intensity-weighed computation, and inflection points to determine boundaries. This step generated LC-MS maps of protein deisotoped masses.

Group volumes were exported to the Analyst module of Genedata Expressionist to perform statistical analyses. Parameters for principal component analysis (PCA) were analysis of rows, covariance matrix, 70% valid values, and row mean imputation. Parameters for hierarchical clustering analysis (HCA) were clustering of columns, shown as tree, positive correlation distances, Ward linkage, and 70% valid values. 

##### Identifications of Cannabis Protein by Mascot

The RAW files were processed in Proteome Discoverer version 2.2 (Thermo Fisher Scientific) as detailed above for the known protein standards to create a single MGF file containing 11,250 MS/MS peak lists. 

The MGF file was searched in Mascot version 2.6.1 (MatrixScience) with top-down searches licence. An MS/MS ion search was performed with the NoCleave enzyme, carbamidomethyl (C) as fixed modification and oxidation (M), acetylation (protein N-term and K), methylation (K), and phosphorylation (ST) as variable modifications, with monoisotopic masses, ±1% precursor mass tolerance, ±50 ppm or ±2 Da fragment mass tolerance, precursor charge of 1+, 9 maximum missed cleavages, and instrument type that accounted for CID, HCD, and ETD fragments (i.e., b-, c-, y-, and z-type ions) of up to 110 kDa. Several databases were searched. The first database searched was the smallest and the most specific and contained all UniprotKB AA sequences from *C. sativa* and close relatives previously used for our BUP (bottom up) study [1], which was updated on 21 August 2019, thus amounting to 663 entries in total (i.e., 73 sequences added in 6 months). The second database searched was the least specific SwissProt *viridiplantae* (39,800 sequences; version 5; last updated 8 March 2019). The third database searched was the largest and was compiled on 21 August 2019 using all the *C. sativa* protein sequences (59,525 in total) from three sources: (1) UniprotKB (663 accessions, https://www.uniprot.org/uniprot/?query=taxonomy:3744%20cannabis%20sativa), (2) NCBI (1451 accessions, https://www.ncbi.nlm.nih.gov/protein/?term=(cannabis+sativa)+AND+%22Cannabis+sativa%22%5Bporgn%3A__txid3483%5D), and (3) the Medicinal Plant Genomic Resource (MPGR) (57,411 accessions, http://medicinalplantgenomics.msu.edu/pub/data/MPGR/Cannabis_sativa/). The decoy option was selected. The error tolerant option was tested as well but was not pursued, as search times proved much longer and number of hits diminished. For the MPGR hits that remained unannotated, the AA sequence was retrieved and blasted in UniprotKB (https://www.uniprot.org/blast/).

## 3. Results and Discussion

### 3.1. TDS of Infused Protein Standards

The known protein standards tested were myoglobin (Myo), β-lactoglobulin (β-LG), α-S1-casein (α-S1-CN), and bovine serum albumin (BSA), which vary not only in their AA sequence and their MW but also in the number of disulfide bridges and PTMs they present. Only mature AA sequences, i.e., not including initial methionine residues and signal peptides, are used for sequencing annotations. Myoglobin (P68083., 153 AAs) can carry a phosphoserine on its third residue, β-lactoglobulin (P02754, 162 AAs) has two disulfide bonds, α-S1-casein (P02662, 199 AAs) is constitutively phosphorylated with up to nine phosphoserines, and BSA (P02769, 583 AAs) contains 35 disulfide bonds as well as various PTMs, most of which are phosphorylations. Oxidation of methionine residues of protein standards was encountered, possibly resulting from vortexing during the sample preparation. Precursors of oxidized proteoforms were purposefully disregarded in the manual annotation step; however, they were included as a dynamic modification for the Mascot search.

Tandem MS data from infused known protein standards fragmented using SID, ETD, CID, and HCD were processed either manually in order to include SID data, which are not considered as genuine MS/MS data, or automatically on bona fide MS/MS data only to test whether an automated workflow would successfully reproduce manual searches and therefore could be applied to unknown proteins from cannabis samples (Figure 1). For this labour-intensive and time-consuming manual curation process, only MS/MS data that corresponded to a match against a major isoform were used. Those corresponding to modified proteoforms such as oxidised myoglobin were ignored. 

#### 3.1.1. Different Fragmentation Modes Produce Different Spectral Patterns in a Precursor-Dependent Manner

**Figure 2:**
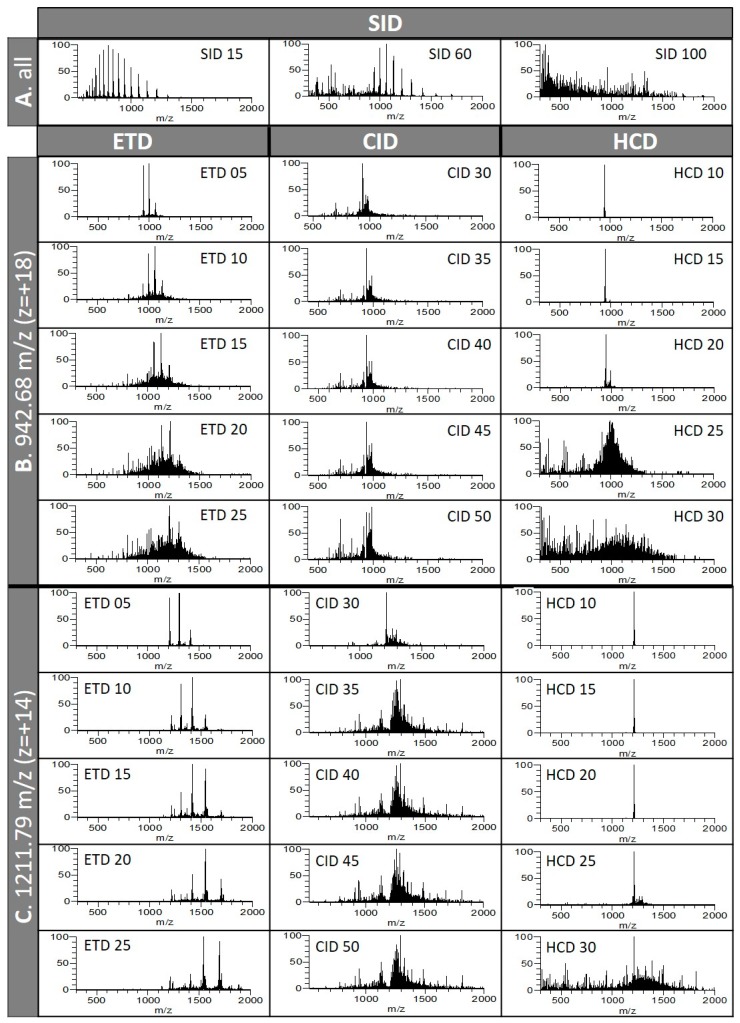
Fourier-transform Orbitrap (FTMS) and FTMS/MS spectra from infused myoglobin. (**A**) Fragmentation of all ions by source-induced dissociation (SID); (**B**) fragmentation of ion 942.68 *m/z* (z = +18) by electron transfer dissociation (ETD), collision-induced dissociation (CID), and higher-energy collisional dissociation (HCD); (**C**) fragmentation of ion 1211.79 *m/z* (z = +14) by ETD, CID, and HCD.

Myoglobin was used to illustrate that the precursor charge state influences the MS/MS spectral pattern, regardless of the fragmentation type; this observation holds true for each standard studied here. Figure 2 displays spectra from myoglobin acquired following SID, ETD, CID, and HCD where increased energy was applied. No fragmentation was observed at SID 15 V. Fragmentation of the most abundant ions of lower *m/z* started to occur at SID 45 V (not shown), was evident at SID 60 V, and completed at SID 100 V (Figure 2A). 

Whilst MS/MS spectra of the most abundant multiply-charged ions were obtained as attested in Table 1, only two charge states, 942.68 *m/z* (z = +18) and 1211.79 *m/z* (z = +14), are exemplified in Figure 2B,C, respectively. Applying ETD for increasingly longer periods, from 5 to 25 ms, resulted in greater protein fragmentations. As ETD fragmentation improved, the fragments’ mass range extended from intermediate to high *m/z* values (Figure 2B). Less fragmentation was observed when ETD was applied for 5 ms (356 and 143 deisotoped fragments for 942.68 *m/z* and 1211.79 *m/z*, respectively) than when ETD was sustained for longer activation times (Table 1). 

The maximum number of fragments was obtained with 20 ms for 942.68 *m/z* (516 deisotoped fragments) and 15 ms from 1211.79 *m/z* (455 deisotoped fragments) (Table 1). In our study, compiling all ETD fragment masses together in Prosight Lite program yielded a myoglobin sequence coverage of 54%. Various proteins comprising myoglobin were analysed previously using an AmaZon ETD mass spectrometer offering an ETD/PRT (proton transfer reaction) option and applying 50 ms for ETD and 100 ms for PRT to charge state +19 covered 85% of myoglobin sequence [32]. Using the newest generation of quadrupole Orbitrap linear ion trap Tribrid hosting a high capacity ETD mode, Riley and colleagues compared the standard ETD mode we used to high capacity ETD (also called EThcD) mode using three known proteins, including myoglobin [33]. High capacity ETD accumulated parent cations in the centre section, allowing larger precursor populations for increased product ion S/N. Monitoring three charge states of myoglobin (z = +21, +18, +15), maximums of 49% and 63% AA sequence coverages were achieved using standard ETD and EThcD, respectively. The authors show that higher charge states of myoglobin were more amenable to ETD fragmentation and that averaging more transient improved S/N and therefore sequencing. In a follow-up study, the same group employed activated ion-electron transfer dissociation (AI-ETD), a method that leverages concurrent infrared photoactivation to enhance electron-driven dissociation, and was able to achieve 65% sequence coverage for charge state +22 of myoglobin [34]. Another team of researchers employed a custom-built high-field FT-ICR mass spectrometer, which enables high-level interrogation of intact proteins in the most detail to date, with the integration of a front-end ETD (FETD) to top-down sequence various proteins, which included myoglobin [35]. The authors show that increasing the cumulative ion (AGC) target led to increased sequence coverage, albeit in a protein size-dependent fashion; the larger the protein is, the greater the AGC target needs to be. They were also able to achieve 65% AA sequence coverage of myoglobin with a 3.0 × 10^6^ AGC target. Aware of this phenomenon, in our study, we chose not to test different AGC target values and rather employed an intermediate value of 1.0 × 10^6^ that suited small to mid-size proteins.

Increasing the energy of CID mode from 35 to 50 eV had less impact on fragmentation, as can be visually assessed in Figure 2B,C and in Table 1, with more constant numbers of fragments generated, albeit still increasing with the energy levels applied. As CID fragmentation intensified, more ions of low *m/z* appeared (Figure 2B). The fewest numbers of fragments were obtained at CID 35 eV (194 and 241 deisotoped fragments for 942.68 *m/z* and 1211.79 *m/z*, respectively), and maximum numbers were reached at CID 50 eV with 209 and 402 fragments for 942.68 *m/z* and 1211.79 *m/z*, respectively (Table 1). In our study, compiling all CID fragment masses together in Prosight Lite program yielded a myoglobin sequence coverage of 44%. Similar to ETD, fragmentation resulting from HCD mode was enhanced as more energy was applied, from 10 to 30 eV. This is clearly visible on Figure 2B,C, with only a handful of fragments observed at HCD 10–15 eV and fragmentation fully developing at HCD 20 eV and above. As HCD fragmentation improved, the mass range of the ions visibly extended (Figure 2B,C). Only 116 and 60 deisotoped fragments were detected at HCD 10 eV from 942.68 *m/z* and 1211.79 *m/z*, respectively, with number of fragments peaking at HCD 25 eV to 511 and 529 for 942.68 *m/z* and 1211.79 *m/z*, respectively (Table 1). In our study, compiling all HCD fragment masses together in Prosight Lite program yielded a myoglobin sequence coverage of 57%. The fact that the outcome of fragmentation was much less dependent on a particular collisional value for CID than for HCD was also noted by Shliaha and colleagues [25]. Futhermore, they report that, while CID and HCD spectra are very similar, HCD achieves optimal fragmentation at lower energy levels, which we also observed in our study. Riley and colleagues indicate that HCD yields the least sequence coverage for most precursors of myoglobin and other known proteins, with its best performance usually occurring for the lowest charge-state parent ions [34]. While we also observed that lower charge states of myoglobin responded better to HCD dissociation, we report the highest sequence coverage using HCD data, which contradicts the authors’ observations.

The present study reveals that different precursors of the same protein (i.e., different charge states) required different energy level for optimum fragmentation, as evidenced in Table 1. It also shows that targeting a lower charge state shifted the fragment masses to the right of the mass range towards high *m/z* values (Figure 2C). Row averages of fragments across all five charge states of myoglobin (+20, +19, +18, +14, +13) listed in Table 1 highlight that a minimum energy level needed to be reached for any meaningful protein dissociation to occur. As far as myglobin is concerned, these values were 60 eV for SID, 25 eV for HCD, 20 ms for ETD, and 40–50 eV for CID, sorted in decreasing order. Column averages of fragments across all MS/MS modes indicate that some precursors were more amenable to fragmentation than others, with charge states +18 (942.68 *m/z*) and +14 (1211.79 *m/z*) on average generating most fragments (325 and 331, respectively, Table 1). This suggests that parent ions displaying both high *m/z* (low charge state) and high intensity should be favoured for top-down sequencing experiments. Shliaha and colleagues demonstrate that targeting different charge states provides complementary information; lower charge states respond well to CID and HCD, and higher charge states are more amenable to ETD fragmentation [25]. It is worth mentioning that another type of electron-based fragmentation technology—ECD available on a Q Exactive orbitrap mass spectrometer—produced up to 48% sequence coverage of myoglobin [36]. Using a hybrid quadrupole FT-ICR mass spectrometer, ECD was demonstrated to outperform CID, particularly on myoglobin [37].

#### 3.1.2. The Central Part of a Protein is Difficult to Fragment, Therefore Recalcitrant to Top-Down Sequencing

Again, we exemplify this observation using myoglobin; however, it was noted on all standards analysed here. All the deconvoluted and the deisotoped masses obtained by applying increasing energy levels of SID, CID, HCD, and ETD were submitted to ProSight Lite and searched against the AA sequence of myoglobin, excluding the initial methionine, which was processed out during the protein maturation step. All the resulting matching b-, c-, y-, and z-type ions are reported in Table 2 and plotted according to their position along the mature AA sequence of myoglobin (153 AA).

**Figure 3:**
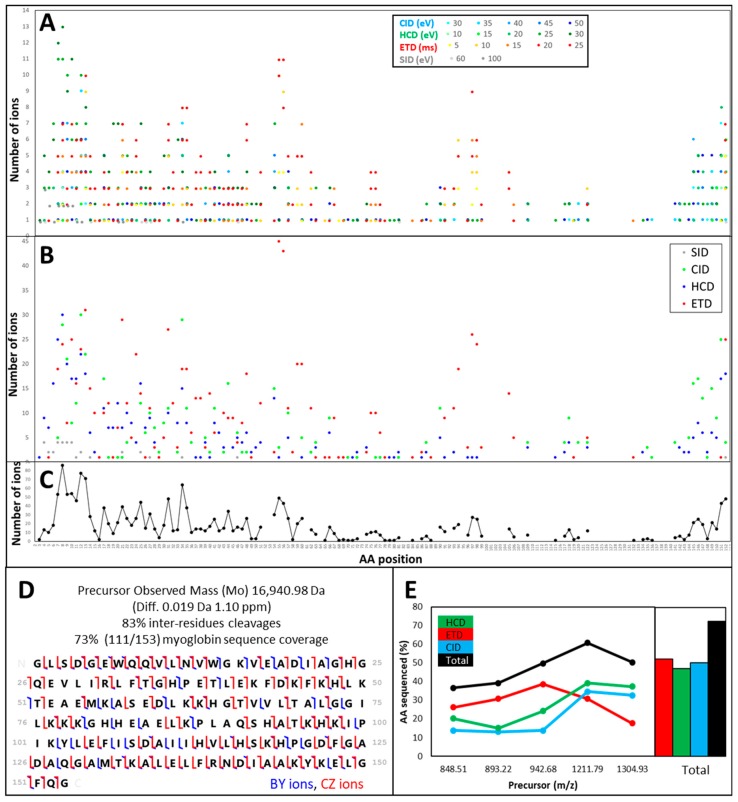
Summary of the number of matching ions in Prosight Lite program achieved for myoglobin. (**A**) Depicting the number of matched ions for every MS/MS parameter tested summed across all five charge states listed in Table 1; (**B**) summed by MS/MS mode along myoglobin amino acid (AA) sequence; (**C**) summed globally across all the data obtained for myoglobin along its AA sequence; (**D**) global AA sequence coverage when all MS/MS data are considered; (**E**) percentage of sequence coverage achieved for each of the five myoglobin charge states.

Because different ions of the same protein underwent different types of fragmentation at varying energy levels, the data are quite redundant, as can be seen in Figure 3A, with many dots depicted at a particular AA position. Higher energy levels produced the most meaningful data, as attested by the prominence of darker shades.

Figure 3B corresponds to the summation of the number of matched ions per MS/MS mode, irrespective of the energy applied. It shows that some parts of the sequence were highly amenable to specific dissociation modes. For instance, ETD was more suited for the N-terminus and the central part of the protein, while CID and HCD helped sequence the C-terminus. CID generated predominantly low yield N- and C-terminal fragments from intact proteins [38]. SID was only effective on the N-terminus of myoglobin. Using an FT-ICR instrument, Cobb and colleagues tested increasingly different SID conditions, also called declustering potentials, from 60 to 240 V on ubiquitin, myoglobin, and BSA [39]. The authors demonstrate that protein dissociation mechanisms are found to be modulated by both source declustering potential and precursor ion charge state. They also explain the canonical and the non-canonical mechanisms involved and how certain AAs such as Pro and Asn are more amenable to C-terminal fragmentation while other AAs such as Ile, Leu, and Ser tend to cleave at the N-terminus of the protein. Like us, their results show that higher SID potentials yield more fragments [39]. Figure 3C represents a summation of the number of matched ions at each AA position, irrespective of the MS/MS mode or the energy applied. Where fewer dots are displayed, the areas of myoglobin that resisted fragmentation under our conditions became apparent. Myoglobin N-terminus was well covered up to position 99, albeit with some interruptions, whereas the C-terminus was only covered up to the last 10 AAs. The region spanning AAs 100 to 140 of myoglobin was only partially sequenced. Shliaha and colleagues [25] published last year an exhaustive top-down proteomics experiment on infused standards, including myoglobin. The authors indicate that CID and HCD contribute less AA sequence information than ETD, mostly at the protein N and the C-termini, whereas ETD provides coverage throughout the protein sequence. They conclude that, as far as myoglobin is concerned, ETD outperforms CID and HCD.

The ProSight Lite output is shown in Figure 3D and confirms that both N- and C-termini of myoglobin sequence were well covered, with many AAs identified from b-, c-, y-, and z-types of ions. Some AAs could only be fragmented once, either using ETD or HCD. Therefore, resorting to multiple MS/MS modes was essential to maximise top-down sequencing. Overall, 83% inter-residues cleavages were annotated, accounting for 73% (111/153 AAs) sequence coverage of myoglobin (Figure 3D). Figure 3E summarises top-down sequencing efficiency for myoglobin in our experiment. It varied according to the charge state and the dissociation type. Only when all fragmentation data were put together could the highest sequence coverage be achieved.

#### 3.1.3. Fragmentation Efficiency Varies from Protein to Protein in a Size-Dependent Fashion

**Figure 4:**
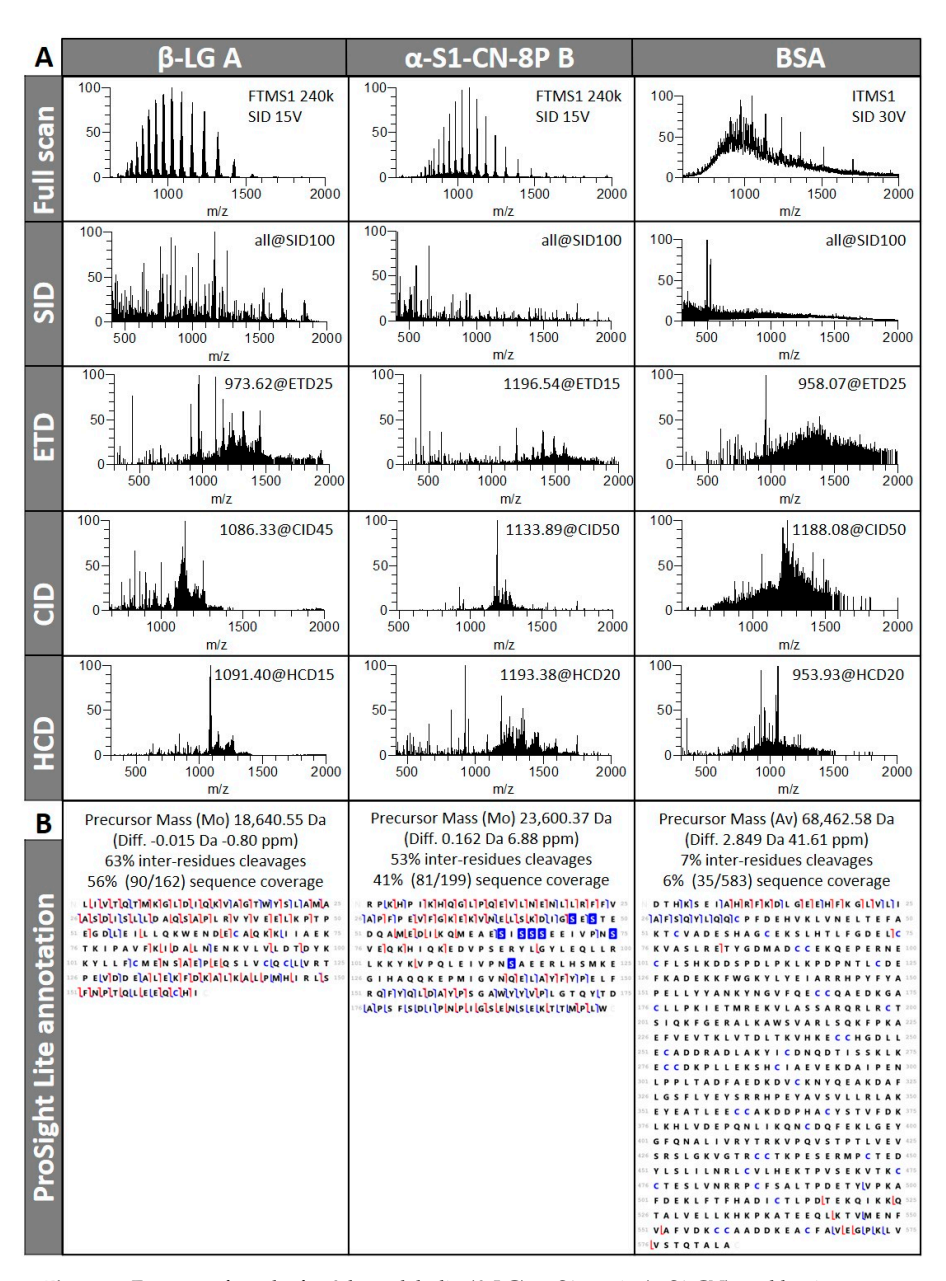
Excerpts of results for β-lactoglobulin (β-LG), α-S1-casein (α-S1-CN), and bovine serum albumin (BSA). (**A**) Examples of FTMS and FTMS/MS spectra using SID, ETD, CID, and HCD; (**B**) global AA sequence coverage when all MS/MS data are considered.

The commercial standards used in this study and past work [28,30] contained mixtures of protein isoforms. Deconvolution of full scan FTMS1 (Figure 4A) supplied accurate masses for β-lactoglobulin, α-S1-casein, and average masses for BSA with an error <50 ppm, which helped in figuring out which protein isoforms underwent MS/MS analysis and which sequence to use for ProSight Lite annotation.

Precursors from allelic variant A of β-lactoglobulin and allelic variant B of α-S1-casein with eight phosphorylations were selected for fragmentation. Examples of SID, ETD, CID, and HCD spectra for each protein can be seen in Figure 4A and illustrate how different they are. Theoretical charge state distributions for proteins showed that the absolute number of charges that precursors carried and the relative width of the charge state distribution both increased as protein mass was augmented [33]. The authors further mention that, as protein size increased, not only did the measureable signal across more fragment ions spread, but also proportionally larger fragment ions with broader isotope distributions were created, thus impacting S/N ratio. In this study, we used a high number of microscans to perform spectral averaging in order to increase S/N, but the tradeoff was a longer duty cycle and acquisition time, which restricted throughput. In previous works, we employed a Q-ToF instrument that offered a spectral summation option and maximized S/N so much that very little background noise was left without overly prolonging the duty cycle [27,28,29]. Another advantage of the Q-ToF system is that it tolerated more complex samples without the need for SPE clean-up, which not only minimised the number of steps during sample preparation but also avoided protein loss. Because we wanted to explore different fragmentation modes, we turned to the LTQ-orbitrap mass spectrometer.

The number of deconvoluted deisotoped fragments of all protein standards is listed in Table 1. As previously observed for myoglobin, fragmentation efficiency assessed on the number of fragments generated depended on the charge state of the precursor, the MS/MS mode, and the energy applied, albeit in a protein-specific fashion. For instance, abundant parents of lower charge states yielded numerous fragments in the cases of β-lactoglobulin (z = +17, 508 fragments on average) and BSA (z = +68, 220 fragments on average), whereas abundant precursor of high charge state yielded numerous fragments in the case of α-S1-casein (z = +21, 406 fragments on average). If we look at which MS/MS mode and which energy level produced the greatest number of fragments on average across all charge states, we find that the ranking for β-lactoglobulin was SID 100 V > HCD 20 eV > CID 35–45 eV > ETD 10 ms. The ranking for α-S1-casein was SID 100 V > HCD 15 eV > CID 35 eV > ETD 10 ms. The ranking for BSA was SID 100 V > ETD 10 ms > HCD 20 eV > CID 50 eV. In a previous top-down study on bovine milk, which included the protein standards used in this work, we showed that higher CID and SID energy levels produce lower *m/z* ions [29]. A greater AA sequence coverage was achieved for β-lactoglobulin and α-S1-casein in that study; retrospectively, an overestimation as the data processing method employed then did not include a decoy validation step and therefore could not eliminate false positives. Using an LTQ-orbitrap instrument and CID fragmentation in the ITMS, a 17% (27/162) AA sequence coverage of β-lactoglobulin was attained [40]. Employing CID and post ion/ion reaction (i.e., in-source decay or ISD, which operates similarly to SID) options offered by MALDI ToF-ToF systems, few fragments were obtained from β-lactoglobulin singly, doubly, and triply-charged precursors, which prevented correct identification of the protein; myoglobin responded better to this type of dissociation and could be identified based on 21 cleavage sites [41]. Suckau and Resemann cleverly exploited MALDI-ToF-ToF capabilities by first producing ISD fragments of intact proteins and subsequently further dissociating them by CID in a pseudo-MS_3_ experiment; they successfully identified BSA using an N-terminus 24-residue sequence tag [42]. More recently, ISD pseudo MS_3_ and pseudo MS_4_ experiments were performed using a MALDI-ToF adapted with a quadrupole ion trap (MALDI-QIT-ToF) instrument to analyse a mixture of known proteins; BSA proved recalcitrant to such strategy [43].

A plethora of fragments does not necessarily translate into high AA sequence coverage, as can be seen when Table 1 and Table 2, similarly arranged, are compared. For instance, under an SID potential of 100 V, α-S1-casein produced 891 fragments, of which only 7 (0.8%) were an AA sequence match. This phenomenon of “overfragmentation” is alluded to in literature [25] and would result from secondary dissociation of the initial daughter ions when normalized collision energies are enhanced. Whilst noticeable for all MS/MS modes tested here, the best evidence of this applied to SID fragmentation with, at best, only 3% (26/656 for myoglobin) of the fragments being annotated in ProSight Lite. Its efficacy in top-down sequencing varied greatly among the proteins studied here, accounting for as little as 1% coverage of BSA sequence, 4% coverage of α-S1-casein sequence, up to 13% for myoglobin and an impressive 41% for β-lactoglobulin (Table 2). Future tests will endeavour at combining SID with genuine MS/MS modes of the Elite LTQ-orbitrap mass spectrometer and developing the processing workflow to computationally analyse the data.

When true MS/MS data resulting from ETD, CID, and HCD experiments are considered, high numbers of fragments are a requisite for proper top-down sequencing, yet it was not the MS/MS spectra with the maximum number of peaks that yielded the greatest number of matched ions in ProSight Lite (Table 1 and Table 2). For instance, in the case of β-lactoglobulin precursor 1091.4 *m/z* undergoing HCD fragmentation, 815 fragments were obtained with 20 eV, which accounted for 29 matched ions, and 608 fragments were obtained with 15 eV, which accounted for 34 matched ions. In another example, this time looking at α-S1-casein precursor 1139.6 *m/z* undergoing CID fragmentations, 35 eV created 455 fragments with only seven being annotated in Prosight Lite, while 435 fragments obtained with 50 eV led to 17 matches. Compiling all fragmentation data obtained for each protein and submitting them to Prosight Lite program gave the maximum sequence coverage achieved in this study: 56% for β-lactoglobulin, 41% for α-S1-casein, and 6% for BSA (Figure 4B). Shen and colleagues also report improved identification success of peptidome and degradome upon using a combination of CID, HCD, and ETD [44]. Maximised protein sequence coverage following multimodal fragmentation was recently confirmed by Shliaha and colleagues in their intricate top-down proteomics study on infused standards ranging from 10–30 kDa [25].

We conclude from our experiments on known proteins of different MWs that sequence coverage varies according to the protein itself, its size (Supplementary Figure S1) and intrinsic properties, the abundance and the charge state of the precursor ion, the MS/MS mode, and the level of energy applied. Therefore, not many general rules can be surmised apart from the fact that complementary MS/MS data yield greater sequence coverage. A key factor, however, is the signal intensity. What was apparent in our study was that precursors of higher S/N generated better fragmentation spectra (not shown). Generally speaking and under our conditions, medium to high energy levels tend to improve sequence annotation. The observations that optimization performed on one protein cannot be extrapolated to other proteins and the highest coverage can only be achieved by optimizing fragmentation for each protein individually were also recently demonstrated [25].

#### 3.1.4. Automatic Workflow Success Depends on Database Searched and Tolerance Parameters

The analysis presented above was exclusively performed manually using the ProSight Lite program. It was very labour-intensive and time-consuming; moreover, it can only be conceived for known proteins such as the standards tested in this study. This is not feasible for complex samples such as plant extracts analysed using an untargeted approach. Therefore, we developed an automated workflow using Proteome Discover to export an MGF containing 371 MS/MS peak lists, which was submitted to the Mascot algorithm. The parameters bearing the greatest impact on the results were tested, namely the database, the type of dynamic modifications, and the fragment tolerance. The search results are summarised in Supplementary Table S1. The Mascot outcome was then compared to our manual curation. The immediate advantage of automation is the speed at which all the data are processed, not accounting for database search times, which can be significant (days if the error-tolerant option is selected in Mascot program). Another advantage is that the search runs in the background, freeing up time to perform other tasks. Finally, automation greatly limits man-made errors.

In the first instance, a homemade database of 59 FASTA sequences [27,28,29] comprising horse myoglobin, all known allelic variants of bovine caseins, and the most abundant bovine whey proteins (α-lactalbumin, β-lactoglobulin, bovine serum albumin) was searched on our local Mascot server using a ±50 ppm fragment tolerance. The Mascot output is reported as a list of proteins and proteoforms in Supplementary Tables S2 and S3, respectively, as well as exemplified in Supplementary Figure S2A. Four accessions are listed based on 105 (28%) MS/MS spectra matched, correctly identifying myoglobin, α-S1-casein variant B, and β-lactoglobulin, albeit not the correct allelic variant. Based on accurate mass and accounting for carbamidomethylation sites, variant A of β-lactoglobulin was expected, and Mascot identified variants E and F instead, which differed at five AA positions due to insufficient sequence coverage. Bovine serum albumin was not identified. Myoglobin achieved the highest score (3782), with 97 MS/MS spectra yielding annotations, 82% of them being redundant, which was expected, as our data were intended to be highly repetitive. Unmodified myoglobin was the most frequently identified (41%), as it was indeed the most abundant proteoform in the spectra. Oxidised proteoforms were also identified in combination or not with phosphorylated and acetylated proteoforms. Six MS/MS spectra led to the correct identification of α-S1-casein B with a score of 123. Several proteoforms were listed, all of them oxidized and bearing from six to 13 phosphorylations. Mascot scores for β-lactoglobulin were below the ion score threshold (<27), indicative of low sequence homology. If the fragment tolerance was increased to ±2 Da, 13 proteins were identified from 322 (87%) MS/MS spectra matches (Supplementary Tables S2 and S3). Search times were in the order of minutes.

In the second instance, all the entries of Swissprot database (559, 228 sequences) were searched with a ±50 ppm fragment tolerance. The Mascot search result is reported in Supplementary Table S2 and Supplementary Figure S2B. Not only was the search much longer than with our smaller, more targeted homemade database (lasting three days), but only myoglobin could be identified based on a total of 46 (12%) matched MS/MS spectra (71% redundancy) and yielding a protein score of 1456. As observed with the homemade database, the unmodified isoform was the most frequently identified (39%), while the other proteoforms bore oxidation and/or phosphorylation sites (Supplementary Table S3). Raising the MS/MS tolerance to 2 Da did not increase the list of proteins identified but brought the score to 8764 with 113 (30%) matches. Limiting Swissprot taxonomy to “other mammalia” brought myoglobin scores to 17,072 with 62 (17%) matches and 10,298 with 136 (37%) matches, respectively, applying ±50 ppm and ±2 Da fragment tolerance. While this reduced search times to hours, it also identified a protein we did not expect in our known protein samples, namely NADH-ubiquinone oxidoreductase, albeit with a low score (46, Supplementary Tables S2 and S3). As the commercial standards we used were not pure [30], it is quite possible that this protein was genuinely present in the sample. We concluded that increasing the search space by choosing a database with more entries and selecting more dynamic modifications lengthened the time needed to complete the search (Supplementary Table S1) without necessarily yielding more relevant identities (Supplementary Table S2).

We cannot explain why using Swissprot as a database failed to identify all the proteins analysed here, namely α-S1-casein, β-lactoglobulin, and BSA. Most likely, the quality of the MS/MS spectra and the signal intensity levels were major contributing factors. This highlights the fact that, with larger databases, meaningful information is lost, likely during the decoy search step. Therefore, specific databases that target the species of interest are better suited for protein sequencing experiments. Using our homemade targeted database proved successful in identifying all the analysed known proteins but the largest (BSA), which not only was challenging to resolve isotopically with the system used in this study but also suffered from low intensity levels and therefore low S/N. Mascot search parameters chosen by Drabik and colleagues imputed different mass tolerances, being more strict (±1.5 Da) at the parent level and less stringent at the product level (±2 Da), which yielded greater score and sequence coverage for myoglobin than in our study [32]. Top down (BIG) Mascot was shown to have limitations by [37]. Mascot consistently provided a higher probability of a false match (i.e., more stringent) compared to the ProSight Lite program. Furthermore, individual internal fragments from large proteins were not confidently matched by Mascot due to, on the one hand, the occurrence of multiple matches, and on the other hand, mass shifts due to PTMs. The authors conclude that the stringency level of Mascot was excessive [37]. Mascot and Open Mass Spectrometry Search Algorithm (OMSSA) algorithm were compared for top-down sequencing purposes [45]. OMSSA identified a larger number of spectra than Mascot; OMSAA displayed better sensitivity and specificity than Mascot. Mascot and OMSSA were then compared to MS-Align+, which significantly yielded a greater number of protein identities [46]. Therefore, it would be worthwhile to test other algorithms than Mascot to prospect top-down proteomics data.

Our tests on protein standards only aimed at reproducing what had been achieved by others in the literature [25,40,44] in order to validate our MS/MS methods; we did not set out to unravel novel information on these standards, which would have been off topic. We successfully used the various fragmentation modes offered by the LTQ-orbitrap mass spectrometer (SID, CID, HSD, and ETD) to top-down sequence known proteins of various sizes. The second part of the study aimed at applying these MS/MS methods to unknown proteins from complex plant samples.

### 3.2. TDS of Cannabis Proteins

Satisfied with our tests on known protein standards, we then analysed protein extracts from cannabis mature buds. Extracts were concentrated by evaporation to maximise signal intensity. The chromatographic separation of intact denatured proteins was fine-tuned (not shown) from 15 to 40% of mobile phase B for 87 min. We decided to not pursue the SID declusturing method and instead only applied ETD, CID, and HCD in succession with three levels of energy, called “low” (ETD 5 ms, CID 35 eV, HCD 19 eV), “mid” (ETD 10 ms, CID 42 eV, HCD 23 eV), and “high” (ETD 15 ms, CID 50 eV, HCD 27 eV).

#### 3.2.1. LC-MS and LC-MS/MS Patterns of Cannabis Protein Extracts are Very Reproducible

**Figure 5:**
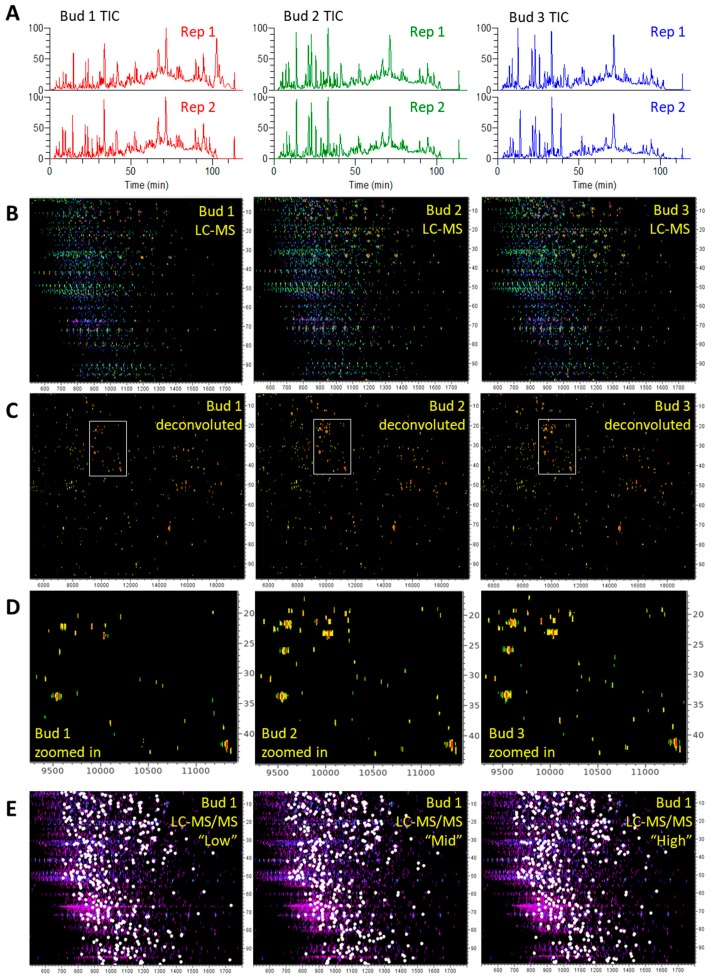
Profiles of medicinal cannabis protein samples. (**A**) Total ion chromatograms (TIC) for each biological replicate (buds 1 to 3) and technical duplicate (reps 1 and 2). Elution times (min) are along the x-axis, and the signal intensity is along the y-axis; (**B**) LC-MS pattern of each biological replicate (buds 1 to 3) (rep 1). Mass range (500–2000 *m/z*) is along the x-axis, and elution times (min) are along the y-axis; (**C**) Deconvoluted LC-MS map of each biological replicate (buds 1 to 3) (rep 1) along 3–30 kDa on the x-axis; (**D**) zoom-in the area boxed in (C) along 15–45 min and 9–11.5 kDa corresponding to abundant proteins; (**E**) triplicated LC-MS/MS patterns from biological replicate bud 1; dots represent MS/MS events.

The three cannabis extracts (buds 1 to 3) were run using LC-MS in duplicate and using LC-MS/MS in triplicate with high reproducibility (Figure 5).

Total ion chromatograms (TIC) were very similar across technical replicates as well as among biological replicates 2 and 3 (Figure 5A); sample bud 1 differed slightly, mostly due to lower signal intensities during the first half of the LC run. LC-MS patterns were very similar, generally differing in peak intensities across biological replicates (Figure 5B), as the number of protein groups was consistent with small standard deviation (SD) values (470 ± 17 groups) (Table 3A).

Maps of deconvoluted masses were also highly comparable, with a greater majority of proteins (93%) being smaller than 20 kD (Supplementary Figure S3 and Figure 5C); a zoom-in confirmed the lesser intensity of bud 1 pattern (Figure 5D). If we compare these LC-MS patterns with those we previously published ([1]), the most obvious difference is the disappearance of the late eluting compounds of low *m/z* values. These abundant singly-charged compounds were eliminated during the clean-up SPE we added in the present study. Other improvements included increasing the chromatographic separation from 60 to 120 min and using UPLC column packed with a C4 rather than a C8 stationary phase. This resulted in better utilization of the 500–2000 *m/z* range (503–1799 *m/z*), enhanced dynamic range (from 10^4^ to 10^8^, i.e., four orders of magnitude), increased numbers of multiply-charged ions, and overall superior and more reproducible LC-MS profiles.

The triplicated LC-MS/MS patterns were also very similar, as exemplified in bud 1 in Figure 5E. Table 3B lists the number of MS/MS spectra per sample (1160 to 1220 MS/MS spectra on average) and method (1178 to 1189 MS/MS spectra on average); SD values were very small and comparable across samples (±8 to 11) and methods (±22 to 31), indicative of high repeatability. The reproducibility of the LC-MS and the LC-MS/MS analyses was statistically assessed (Supplementary Figure S4). PCA clearly separated LC-MS data from LC-MS/MS data along Eigenrow 1 and bud 1 sample from the other two biological samples along Eigenrow 2. Technical replicates were clustered together. This was confirmed by HCA.

#### 3.2.2. Proteins from Cannabis Buds are Small

The most abundant multiply-charged precursors targeted for MS/MS fragmentation are listed in Table 4, which also highlights some of their features.

Overall, precursor charge states ranged from +2 to +25, parent ions from 700.4094 to 1729.6853 *m/z*, and their accurate masses spanned 1426.3553 to 25,389.9953 Da. Inherent to MS, the greater the charge state was, the greater the mass of cannabis proteins was (Supplementary Figure S5A). The most abundant precursors bore four to 10 charges, and their accurate masses ranged from 2.8 to 17.3 kDa. Therefore, this type of analysis predominantly favoured small proteins from cannabis buds.

Another factor determining precursor selection pertains to protein abundance, emulated by base peak intensity in the mass spectrometer. Indeed, for a protein larger than 20 kDa to undergo MS/MS, its base peak intensity must exceed 2000 counts (Supplementary Figure S5B).

The last factor determining precursor selection relates to protein hydrophobicity, which affects the chromatographic elution. Supplementary Figure S5C reveals that proteins larger than 20 kDa eluted after 75 min of reverse phase separation; therefore, they must have been more hydrophobic than proteins of smaller size. This means that, for highly hydrophobic proteins, the separation method would need refining by using a different type of stationary phase and/or different mobile phases and gradients.

#### 3.2.3. The Vast Majority of MS/MS Data from Cannabis Samples Remains Unannotated

A total of 11,250 MS/MS peak lists were searched against the UniprotKB *C. sativa* database (663 entries) using the Mascot algorithm, a fragment tolerance of ±50 ppm or ±2 Da, and validating the results using a decoy or an error tolerant method (Supplementary Table S1). With a ±50 ppm fragment tolerance, protein N-term acetylation and Met oxidation set as dynamic modifications, and an error tolerant method, 12 proteins were identified [210 (2%) matches] with 11,040 (98%) MS/MS spectra remaining unassigned and a search time of over 24 h. Using the same parameters but changing error tolerance to decoy brought the number of accessions identified to 21 from 213 (2%) matched MS/MS spectra and a very fast search time of 29 s (Table 5). Excessive stringency in Mascot algorithm could justify the low number of database hits [37], as discussed above in the sections pertaining to known protein standards. Relaxing the fragment tolerance to ±2 Da listed 36 proteins based on 355 (3%) assigned MS/MS spectra with a search time of 2.5 min. With a ±50 ppm fragment tolerance, protein N-term acetylation, Met oxidation, and phosphorylations of Ser and Tyr residues set as dynamic modifications and a decoy method, the number of unique proteins identified was 21 (187 matches) over a search time of 2 h. Lifting the fragment tolerance to ±2 Da increased the number of hits to 61 proteins with 590 (5%) MS/MS spectra assigned. Forsaking dynamic modification reduced search times to mere seconds and yielded 20 and 24 identities using ±50 ppm and ±2 Da fragment tolerance, respectively (Supplementary Tables S1 and S4).

To further evaluate the effect of Mascot search parameters, a more extensive but less targeted database was interrogated using the least stringent fragment tolerance (±2 Da) and a decoy method. We chose Swissprot, as it is a curated database currently hosting over 200 million sequences. Without any dynamic modification set, searching the whole taxonomy yielded 94 accessions with 998 (9%) MS/MS matches, and searching only *viridiplantae* taxonomy (39,800 entries) yielded 80 hits [1181 (10%) matches]. Searching *viridiplantae* taxonomy and setting protein N-term acetylation and Met oxidation as dynamic modifications listed 141 accessions [1352 (12%) matches]. Finally, still searching *viridiplantae* taxonomy but adding phosphorylations of Ser and Tyr residues as dynamic modification generated 274 accessions [1863 (17%) matches]. The latter search lasted the longest (53 h) (Supplementary Tables S1 and S4). While the list of proteins extends when a larger database is used in conjunction with more relaxed mass tolerances, we do not yet believe in their relevance, as only one protein (Olivetolic acid cyclase, OAC) actually comes from *C. sativa* species. Previously, Shen and colleagues demonstrate that more decoys fragments (i.e., false positives) are identified when the mass error increases [44]. Furthermore, lower mass accuracy decreases the specificity of identification [37]. For these reasons, we hereafter only focus on the search result obtained from the uniprotKB database with a stringent fragment tolerance (±50 ppm) (Table 5).

We then searched a larger and more specific database containing all the *C. sativa* protein sequences we could retrieve from UniprotKB, NCBI, and MPGR, which amounted to a total of 59,525 accessions. Running the Mascot search with a 50 ppm tolerance and no modifications was very quick (1.2 min) and yielded 31 accessions (Table S1). However, imputing variable modifications significantly augmented the search time. For instance, searching for oxidation (M) and N-term acetylation lasted 1 h and produced 36 accessions; search for methylation (K) lasted 2 h and yielded 33 accessions. Anything else turned into day-long searches (Table S1). Setting modifications exponentially increased the search space, thereby exponentially increasing the duration of the search. To overcome this issue, we devised a two steps strategy: (1) In Mascot, we minimized the number of PTMs (none or oxidation M) in order to quickly generate a list of hits. Not including the search outcomes using the second database (Swissprot *viridiplantae*), a total of 46 unique accessions were identified in medicinal cannabis mature buds bearing numerous PTMs (Table 5); (2) in the Genedata Refiner LC-MS maps of the deconvoluted masses, we explored features in the vicinity of these hits to detect more proteoforms. This allowed the detection of 136 proteoforms (Supplementary Table S5).

NCBI yielded one (2%) accession (AKP55264.1 30S ribosomal protein S16, chloroplastic), MPGR yielded 20 (43%) accessions, and UniprotKB yielded 25 (55%) accessions. This suggests that, even though UniprotKB hosts the smallest number of *C. sativa* accessions (663) relative to NCBI (1451) and MPGR (57,411), it generated the highest number of identities, a testament to UniprotKB quality and well-deserved status of reference database for proteins. The masses of the 46 identified proteins ranged from 3.8 kD to 17.9 kD. Twenty accessions had a Mascot score above 100, and 36 accessions were identified using more than one MS/MS spectrum (Table 5 and Supplementary Table S5). No missed cleavage was found (M > 0), possibly explaining the low number of identified proteins, as we were expecting natural protein degradation to occur. The protein identified with the highest score was cytochrome b559 subunit alpha (accession A0A0C5ARS8, score of 1641, 29 matches, Supplementary Figure S6). Only nine (20%) of the intact proteins identified by TDP were also identified in our BUP study [1], which highlights the complementarity of both approaches.

**Figure 6:**
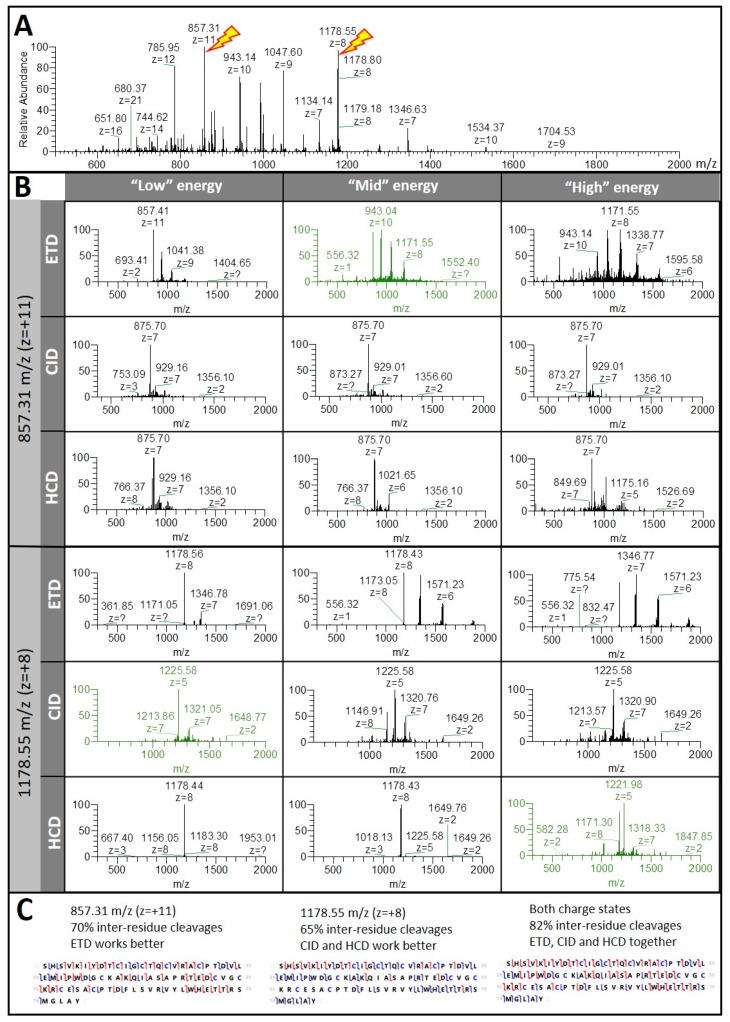
Top-down sequencing summary for *C. sativa* Photosystem I iron-sulfur centre (PS I Fe-S centre, accession A0A0C5AS17). (**A**) FTMS spectra at 30.8 min, lightning bolts depicts the two most abundant charge states chosen for MS/MS fragmentation; (**B**) FTMS/MS spectra for both charge states using each of the three MS/MS methods; spectra in green represent the energy level for a particular MS/MS mode that yielded the best sequencing information; (**C**) AA sequence coverage for each of the charge states and then combined.

As previously observed on the protein standards, fragmentation efficiency of cannabis intact proteins depends on the charge state of the parent ion, on the type of MS/MS mode, and on the level of energy applied. We illustrated this using the protein exhibiting the second highest Mascot score (1664), Photosystem I iron-sulfur centre (PS I Fe-S centre, accession A0A0C5AS17), identified with 39 MS/MS spectra. Fragmentation efficiency was assessed using the ProSight Lite program by the percentage of inter-residue cleavages achieved. MS/MS spectra differed in the number of peaks and their distribution along the mass range (Figure 6A,B).

The optimum dissociation of a precursor ion with high charge state (857.31 *m/z*, z = +11) was achieved with ETD at “mid” energy, whereas a precursor ion of comparable intensity but with lower charge state (1178.55 *m/z*, z = +8) responded better to CID and HCD at “low” and “high” energy levels, respectively. All MS/MS data considered, fragmenting 857.31 *m/z* and 1178.55 *m/z* parent ions yielded 70% and 65% inter-residue cleavages, respectively, and 82% all together (Figure 6C). In order to maximise AA sequence coverage, it was essential to multiply the MS/MS conditions on as many precursor ions as possible. This, of course, limited the total number of different proteins analysed in a top-down approach. Coupling this strategy with an extended separation run should alleviate this drawback.

Most of the identified proteins (17/46, 37%) were involved in photosynthesis (subunits of cytochromes and photosystems I and II, as well as chloroplastic ATPases), then in protein translation (eight ribosomal proteins, 17%). Also identified were six histones. Only one protein belonged to the phytocannabinoid biosynthesis, olivetolic acid cyclase (I6WU39, OAC), previously identified by BUP [1,17]. With 46 identities, this TDP experiment identified fewer individual proteins than our previous shotgun study, which produced 160 accessions using a database from UniproKB [1]; however, the power of TDP lies in the detection of proteoforms of the identified proteins (Table 5 and Supplementary Table S5). In particular, the N-terminus excision of the initial M (NME, Table 5) was determined for 28 (61%) accessions. This valuable and novel information is not found in public databases such as UniprotKB. The PTMs identified in this work were methylation (K), dimethylation (K), acetylation (N-term and K), succinylation (K), phosphorylation (STY), as well as oxidation (M). Seventeen (37%) proteoformes were oxidized (O, Table 5). Our experimental design did not allow for distinguishing between in vivo protein oxidation and artefactual oxidation occurring during sample preparation. Ursem and colleagues demonstrated that centrifugation, freeze-thaw cycles, or long-term storage at −20°C or −80°C did not oxidise proteins [47]. The authors did not test the effect of sample agitation such as vortexing, for instance, whereby aerating the sample can trigger spontaneous oxidation. This might have occurred in our experiment. If needed, a method is available to eliminate this analytical artefact [48]. Limiting the number of steps in sample preparation lowers the chance of artefactual oxidation. A critical review of this approach has been undertaken and documented [49].

Eighteen (39%) proteoforms bore an N-terminus acetylation (NA, Table 5). N-terminal acetylation corresponded to the appending of an acetyl group to the N-terminal amino group in an irreversible manner, which impacted the lifespan, the folding characteristics, and the binding properties of the acetylated protein [50]. This is a widespread protein modification across all taxa, and *C. sativa* is no exception, as demonstrated in this work. Another fairly frequent PTM in our dataset was the methylation of K residues reported in eight (17%) protein accessions (M, Table 5). The post-translational methylation of K residues catalysed by lysine (K)-specific protein methyltransferases (KMTs) is a very common and important protein modification [51] and is particularly well documented for histones with respect to DNA processing [52]. While we did not identify any KMTs by BUP [1], we suspect they are present and highly active in cannabis buds, given the frequency of methylated proteoforms (Table 5). We identified four accessions with phosphorylations (P, Table 5). Phosphoproteins are ubiquitous to all kingdoms; they have been well documented in human, mice, and yeast but much less in *Arabidopsis* [53], the reference plant. Plants contain glycoproteins; only a few reports have applied MS to profile intact plant N-glycoproteins, and their analysis involves specific technical steps, such as the deglycosylation of glycoproteins and their recovery by lectin-affinity columns [54], which were not attempted here. However, this warrants future investigation.

**Figure 7:**
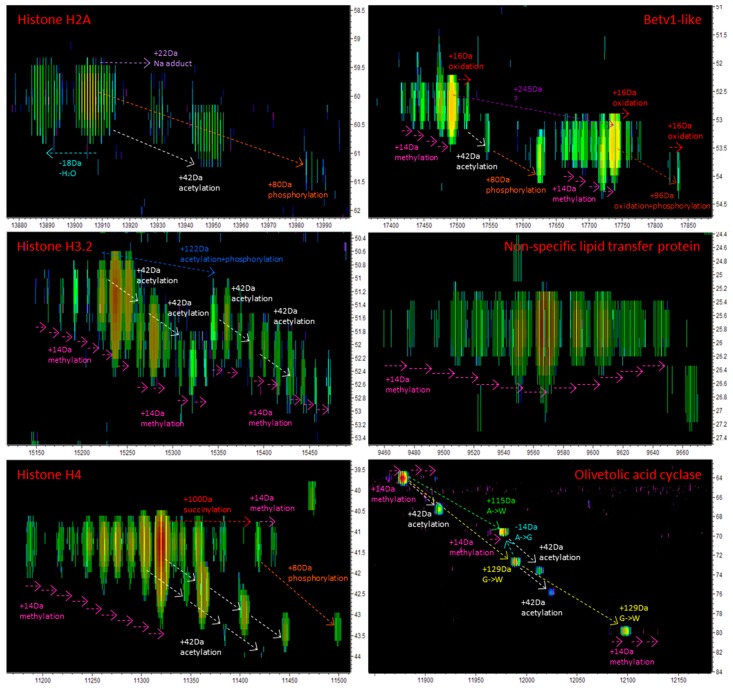
Examples of PTMs of cannabis proteins identified by top-down proteomics (TDP).

Examples of proteoforms detected in this TDP study are given in Figure 7.

Histones were heavily modified [55]—in particular, they were heavily methylated [52]—which we demonstrate in Figure 7 on histones H3.2 and H4. Histones were also acetylated and phosphorylated [56], as shown for histones H2A, H3.2, and H4 (Figure 7). We also found a succinylated proteoform of histone H4. A non-specific lipid transfer protein (nsLTP) also exhibited several methylated states (Figure 7), which has never been shown before. Due to their high allergenicity and cross-reactivity, nsLTPs are major allergens of *C. sativa* [57]. Perhaps some methylated proteoforms of nsLTP are more allergenic than other. Bet v1-like protein (UniprotKB accession I6XT51) is similar to the major pollen allergen Bet v 1. In our sample, we found this protein to be methylated, acetylated, oxidized, and phosphorylated (Figure 7). We also observed that the same group of proteoforms shifted by 245 Da, which we could not assign to any obvious modification. With a Mascot score of 174, OAC was identified without its initial M residue, which has not been evidenced before. Several methylated and acetylated proteoforms of OAC WEre identified (Supplementary Table S5 and Figure 7), along with a possible allelic variant (Figure 7). However, the latter must be confirmed by top-down sequencing.

## 4. Conclusions

This was the first top-down MS proteomics study on medicinal cannabis reproductive tissues. In this work, we tested various MS/MS parameters first on known proteins and then on intact denatured proteins from cannabis buds. Protein fragmentation efficiency depends on the type of MS/MS mode, the level of energy applied, and the charge state of the precursor ion. While some conditions proved less optimal, they still yielded complementary sequencing information. Consequently, the more MS/MS data were acquired for a given protein, the greater the AA sequence coverage was. The biggest limitation was the size of the protein with greater sequence coverage observed for smaller molecules. Data analysis proved challenging, and most of the MS/MS spectra remained unannotated for a reason yet to be elucidated. Prosight Lite program yielded the most exhaustive sequencing information but only operates on known proteins. We found that the best strategy to explore cannabis top-down data was to first search MS/MS data using Mascot with a limited number of PTMs and stringent tolerances followed by the exploration of LC-MS deconvoluted maps in Genedata Refiner to further detect proteoforms. Future work will involve further fine-tuning of the data processing and applying this top-down strategy to different cultivars of medicinal cannabis with the hope of discovering more allelic variations and PTMs.

## Figures and Tables

**Table 1:** Number of spectral MS/MS fragments for each protein standard, each MS/MS mode, and energy parameters. The higher the number of fragments is, the darker the colour is.

			MS/MS Mode	SID	SID	SID	CID	CID	CID	CID	CID	ETD	ETD	ETD	ETD	ETD	HCD	HCD	HCD	HCD	HCD	min	max	mean
			NCE	15	60	100	30	35	40	45	50	5	10	15	20	25	10	15	20	25	30			
Protein	*m/z*	z	RI (%) ^1^																					
Myoglobin	all	n.a.	n.a.	171	725	656																171	656	517
Myoglobin	848.51	20	100				210	255	223	226	233	220	66	120	135	89	102	146	250	253	303	66	303	189
Myoglobin	893.22	19	98				174	180	176	219	227	229	172	190	457	431	71	148	244	301	260	71	457	232
Myoglobin	942.68	18	96				194	233	243	227	209	356	470	504	516	468	116	175	280	511	376	116	516	325
Myoglobin	1211.79	14	38				241	369	389	385	402	143	392	455	411	365	60	105	252	529	462	60	529	331
Myoglobin	1304.93	13	24				180	389	411	383	368	79	282	273	309	263	42	118	262	499	572	42	572	295
Myoglobin	mean			171	725	656	200	285	288	288	288	205	276	308	366	323	78	138	258	419	395			274
b-LG A	all	n.a.	n.a.	543	2160	3882																543	3882	2195
b-LG A	972.19	19	46				336	392	333	358	343	379	375	325	412	242	155	395	504	310	298	155	504	344
b-LG A	1026.15	18	74				344	412	397	439	387	220	271	137	170	102	230	469	588	449	350	102	588	331
b-LG A	1091.40	17	80				397	507	474	511	440						252	608	815	634	443	252	815	508
b-LG A	1232.84	15	100				481	529	571	531	544	160	456	433	431	443	119	517	664	737	419	119	737	469
b-LG A	mean			543	2160	3882	390	460	444	460	429	253	367	298	338	262	189	497	643	533	378			413
a-S1-CN	all	n.a.	n.a.	414	728	891																414	891	678
a-S1-CN	1139.60	21	94				159	455	401	455	435						112	660	660	431	289	112	660	406
a-S1-CN	1193.38	20	100				166	460	466	389	375	111	424	352	292	193	120	702	651	519	301	111	702	368
a-S1-CN	1319.30	18	70									97	302	224	209	145	51	721	586	544	256	51	721	314
a-S1-CN	1397.14	17	52				51	247	259	254	259											51	259	214
a-S1-CN	1480.59	16	36														46	472	464	459	251	46	472	338
a-S1-CN	mean			414	728	891	125	387	375	366	356	104	363	288	251	169	82	639	590	488	274			324
BSA	all	n.a.	n.a.		84	436																84	436	260
BSA	953.93	72	72									0	161	58	124	58	0	232	238	113	85	0	238	107
BSA	994.98	69	76				0	182	150	153	157						0	196	227	121	87	0	227	127
BSA	1061.50	65	68				0	203	177	196	223	0	359	409	352	277						0	409	220
BSA	1188.08	59	44				0	109	96	101	125											0	125	86
BSA	mean				84	436	0	165	141	150	168	0	260	234	238	168	0	214	233	117	86			145

^1^ RI, relative intensity; BSA: bovine serum albumin, n.a, not applicable.

**Table 2:** Number of matching ions in Prosight Lite program (tolerance of 50 ppm) for each protein standard, each MS/MS mode, and energy parameters. The higher the number of matching ions is, the darker the colour is.

			MS/MS Mode	SID	SID	SID	CID	CID	CID	CID	CID	ETD	ETD	ETD	ETD	ETD	HCD	HCD	HCD	HCD	HCD	min	max	mean	No. AAs	% max
			NCE	15	60	100	30	35	40	45	50	5	10	15	20	25	10	15	20	25	30					
Protein	*m/z*	z	RI (%) ^1^																							
Myoglobin	all	n.a.	n.a.	1	19	20																1	20	13	153	13
Myoglobin	848.51	20	100				10	12	11	10	19	25	17	28	40	28	2	4	9	17	17	2	40	17	153	26
Myoglobin	893.22	19	98				4	8	8	9	12	6	24	17	45	48	3	2	11	11	11	2	48	15	153	31
Myoglobin	942.68	18	96				10	12	14	14	14	17	36	45	57	53	2	5	22	33	22	2	57	24	153	37
Myoglobin	1211.79	14	38				27	42	44	39	36	5	24	29	36	26	1	2	12	48	52	1	52	28	153	34
Myoglobin	1304.93	13	24				13	41	40	44	44	2	21	20	21	19	1	4	7	55	47	1	55	25	153	36
Myoglobin	mean			1	19	20	13	23	23	23	25	11	24	28	40	35	2	3	12	33	30	2	45	20	153	30
b-LG A	all	n.a.	n.a.	2	27	66																2	66	32	162	41
b-LG A	972.19	19	46				11	17	20	20	21	8	20	14	20	20	1	14	19	15	21	1	21	16	162	13
b-LG A	1026.15	18	74				11	18	19	20	17	4	9	9	14	11	6	28	24	22	20	4	28	15	162	17
b-LG A	1091.40	17	80				11	24	23	26	18						5	34	29	28	26	5	29	22	162	18
b-LG A	1232.84	15	100				23	23	21	23	22	4	8	12	13	19	3	17	27	27	21	3	23	18	162	14
b-LG A	mean			2	27	66	14	21	21	22	20	5	12	12	16	17	4	23	25	23	22	3	33	21	162	21
a-S1-CN	all	n.a.	n.a.	1	3	7																1	7	4	199	4
a-S1-CN	1139.60	21	94				4	7	8	7	17						1	24	37	43	37	1	43	19	199	22
a-S1-CN	1193.38	20	100				2	10	9	10	6	3	23	25	24	25	2	32	41	37	36	2	41	19	199	21
a-S1-CN	1319.30	18	70									0	13	15	19	18	1	30	35	39	38	0	39	23	199	20
a-S1-CN	1397.14	17	52				6	12	12	9	15											6	15	11	199	8
a-S1-CN	1480.59	16	36														1	28	33	39	38	1	39	28	199	20
a-S1-CN	mean			1	3	7	4	10	10	9	13	2	18	20	22	22	1	29	37	40	37	2	31	17	199	15
BSA	all	n.a.	n.a.		1	4																1	4	2	583	1
BSA	953.93	72	72									0	6	4	8	7	0	9	13	11	9	0	13	7	583	2
BSA	994.98	69	76				0	4	5	5	1						0	3	11	12	11	0	12	5	583	2
BSA	1061.50	65	68				0	6	5	5	6	0	4	8	4	8						0	8	5	583	1
BSA	1188.08	59	44				0	4	2	3	7											0	7	3	583	1
BSA	mean				1	4	0	5	4	4	5	0	5	6	6	8	0	6	12	12	10	0	9	4	583	2

^1^ RI, relative intensity.

**Table 3:** Statistics on cannabis proteins analysed by LC-MS and LC-MS/MS. (**A**) Number of protein groups obtained from Genedata Refiner analysis of the LC_MS data across the three biological replicates (buds 1 to 3) and two technical replicates (replicate 1 and 2); (**B**) number of MS/MS spectra collected for each biological replicate (bud 1 to 3) across each “low, “mid”, and “high” MS/MS method.

(A)						(B)					
Tech. Rep.	bud 1	bud 2	bud 3	Mean	SD	Method	bud 1	bud 2	bud 3	Mean	SD
Replicate 1	442	483	483	469	19	“Low”	1157	1169	1208	1178	22
Replicate 2	474	486	453	471	14	“Mid”	1173	1193	1226	1197	22
mean	458	485	468	470	17	“High”	1149	1192	1225	1189	31
SD	16	2	15			mean	1160	1185	1220		
						SD	10	11	8		

**Table 4:** Statistics on parent ions from cannabis proteins analysed by LC-MS/MS.

Charge State	No. of Precursors	Min. *m/z*	Max. *m/z*	Min. Mass (Da)	Max. Mass (Da)	No. of MS/MS Events
2	34	714.18	1500.37	1426.36	2998.73	63
3	8	848.75	1176.15	2543.23	3525.44	32
4	45	714.08	1380.06	2852.31	5516.21	143
5	39	803.49	1325.52	4012.42	6622.58	120
6	43	775.62	1458.49	4647.67	8744.89	109
7	61	747.77	1534.29	5227.35	10,732.96	222
8	86	787.70	1429.84	6293.52	11,430.63	341
9	69	700.41	1564.79	6294.62	14,074.01	262
10	48	756.92	1729.69	7559.16	17,286.78	195
11	32	726.96	1338.87	7985.51	14,716.50	113
12	30	710.98	1338.68	8519.65	16,052.07	99
13	32	762.47	1256.51	9898.99	16,321.52	114
14	36	732.89	1318.67	10,246.31	18,447.31	125
15	32	738.60	1099.47	11,063.95	16,433.03	109
16	29	708.10	1153.96	11,269.49	18,447.30	105
17	29	737.28	1129.03	12,516.63	19,176.39	86
18	27	754.89	1163.66	13,569.88	20,927.81	96
19	37	715.21	1135.96	13,569.85	21,564.03	124
20	38	710.24	1240.59	14,184.59	24,791.58	126
21	34	723.89	1185.04	15,180.59	24,864.66	106
22	28	701.95	1155.10	15,420.70	25,390.00	92
23	14	711.74	1104.83	16,346.79	25,387.98	31
24	8	746.08	1036.99	17,881.77	24,863.64	18
25	3	745.98	992.59	18,624.23	24,789.59	3

**Table 5:** List of 46 cannabis proteins identified by top-down proteomics using Mascot algorithm, the first and the third databases, various post-translational modifications (PTMs), ±50 ppm fragment tolerance and ordered by description.

No.	Accession	Score	Mass (Da)	RT (min)	No. of Matches	Description	Species	Database	Proteoform ^1^	BUP ^2^
1	A0A0U2H3S7	111	11721	7.5	4	30S ribosomal protein S14, chloroplastic	Humulus lupulus	Uniprot	NME, O	no
2	AKP55264.1	111	10442	40.5	1	30S ribosomal protein S16, chloroplastic	Cannabis sativa	NCBI		no
3	csa_locus_3973_iso_2_len_767_ver_2	41	15430	18.8	4	40S ribosomal protein S24	Actinidia chinensis	MPGR	NME, NA	no
4	csa_locus_3786_iso_3_len_389_ver_2	345	6796	2.9	3	40S ribosomal protein S30	Dichanthelium oligosanthes	MPGR		no
5	A0A0H3W8B6	21	15440	47.5	1	50S ribosomal protein L16	Cannabis sativa	Uniprot	M	yes
6	A0A0C5ARQ5	180	8020	100.3	9	ATP synthase CF0 C subunit	Cannabis sativa	Uniprot	NA, O	no
7	A0A0C5AUH9	62	14615	75.0	3	ATP synthase CF1 epsilon subunit	Cannabis sativa	Uniprot	NME, NA, O	yes
8	A0A0U2H159	54	14697	75.0	3	ATP synthase epsilon chain, chloroplastic	Humulus lupulus	Uniprot	NA, O, D	no
9	I6XT51	80	17490	52.6	6	Betv1-like protein	Cannabis sativa	Uniprot	NME, NA, O, M, P	yes
10	A0A0C5ARS8	1641	9257	96.4	29	Cytochrome b559 subunit alpha	Cannabis sativa	Uniprot	NME, NA	yes
11	A0A0U2GZT5	902	9237	96.4	1	Cytochrome b559 subunit alpha	Humulus lupulus	Uniprot	NME	yes
12	A0A0C5AUI2	163	4392	71.9	15	Cytochrome b559 subunit beta	Cannabis sativa	Uniprot		no
13	A0A0H3W844	24	17381	99.2	1	Cytochrome b6-f complex subunit 4	Cannabis sativa	Uniprot	NME	no
14	A0A0C5APY4	27	4195	101.1	1	Cytochrome b6-f complex subunit 5	Cannabis sativa	Uniprot		no
15	csa_locus_2489_iso_3_len_603_ver_2	20	14971	35.6	2	Furry	Trema orientale	MPGR	NME, NA	no
16	csa_locus_15285_iso_1_len_577_ver_2	43	8673	27.6	3	GAG1At protein	Trema orientale	MPGR	NME, NA, O	no
17	csa_locus_3395_iso_3_len_637_ver_2	14	10650	31.8	2	GroES-like protein	Corchorus capsularis	MPGR	NME, NA	no
18	csa_locus_4170_iso_1_len_735_ver_2	203	13905	59.9	20	Histone H2A	Morus notabilis	MPGR	NME, A, P	no
19	csa_locus_3458_iso_4_len_603_ver_2	87	14959	47.1	4	Histone H2B	Cicer arietinum	MPGR	NME, O	no
20	csa_locus_1853_iso_2_len_1208_ver_2	17	17895	49.2	1	Histone H3.2	Triticum aestivum	MPGR		no
21	csa_locus_1853_iso_1_len_474_ver_2	44	15234	50.7	5	Histone H3.2	Triticum aestivum	MPGR	NME, M, A, P	no
22	csa_locus_11346_iso_1_len_965_ver_2	143	15352	15.5	10	Histone H3.3	Meleagris gallopavo	MPGR	NME, NA, O, M, A	no
23	csa_locus_61264_iso_2_len_414_ver_2	303	11322	41.5	27	Histone H4	Phanerochaete chrysosporium	MPGR	NME, M, A, P, S	no
24	csa_locus_4104_iso_2_len_741_ver_2	151	17736	53.3	7	Major latex/Bet v I type allergen	Parasponia andersonii	MPGR	NME, NA, A	no
25	csa_locus_2495_iso_2_len_611_ver_2	37	9659	75.0	4	Mitochondrial outer membrane translocase complex, subunit Tom	Trema orientale	MPGR	NME, NA, O	no
26	W0U0V5	28	9563	26.2	2	Non-specific lipid-transfer protein (Fragment)	Cannabis sativa	Uniprot	M	yes
27	csa_locus_1463_iso_3_len_564_ver_2	45	7624	18.7	1	Non-specific lipid-transfer protein AKCS9	Parasponia andersonii	MPGR		yes
28	I6WU39	174	11901	65.9	11	Olivetolic acid cyclase	Cannabis sativa	Uniprot	NME, NA, O, M, A	yes
29	A0A0C5AS17	1538	9415	31.0	39	Photosystem I iron-sulfur centre	Cannabis sativa	Uniprot	NME, O	yes
30	A0A0C5AS04	15	4814	98.9	2	Photosystem I reaction centre subunit IX	Cannabis sativa	Uniprot	O, A	no
31	A0A0C5AS00	30	4094	99.1	2	Photosystem I reaction centre subunit VIII	Cannabis sativa	Uniprot	O, P	no
32	A0A0C5B2J7	1167	7515	99.6	11	Photosystem II reaction centre protein H	Cannabis sativa	Uniprot	NME, O	no
33	A0A0C5APX7	249	4193	100.1	5	Photosystem II reaction centre protein I	Cannabis sativa	Uniprot	NA, O, M	no
34	A0A0C5APY3	66	4193	100.3	1	Photosystem II reaction centre protein J	Cannabis sativa	Uniprot	NA	no
35	A0A0H3W8G1	25	4363	99.2	2	Photosystem II reaction centre protein L	Cannabis sativa	Uniprot	NME	no
36	A0A0U2DTK8	1438	3843	99.7	25	Photosystem II reaction centre protein T	Cannabis sativa	Uniprot		no
37	A9XV92	1243	3843	99.7	18	Photosystem II reaction centre protein T	Cannabis sativa	Uniprot		no
38	A0A0C5AUI5	72	7780	4.5	1	Ribosomal protein L33	Cannabis sativa	Uniprot	NME	no
39	A0A0C5B2H7	53	11720	7.5	2	Ribosomal protein S14	Cannabis sativa	Uniprot	NME, A	no
40	A0A0H3W6G0	82	10443	40.5	3	Ribosomal protein S16	Cannabis sativa	Uniprot	M, A	no
41	csa_locus_3039_iso_2_len_611_ver_2	125	9779	42.5	5	Small zinc finger/mitochondrial import inner membrane translocase subunit TIM10-like	Juglans regia	MPGR	NME, NA, O, A	no
42	csa_locus_13354_iso_1_len_537_ver_2	31	9073	55.7	2	Small zinc finger/Tim10/DDP family zinc finger	Parasponia andersonii	MPGR	O	no
43	csa_locus_2526_iso_1_len_396_ver_2	227	6442	54.2	10	Ubiquinol-cytochrome c reductase complex 6.7 kDa protein	Trema orientale	MPGR	NME	no
44	csa_locus_5849_iso_5_len_634_ver_2	178	13600	31.1	4	Uncharacterized protein	Fagus sylvatica	MPGR	NME, NA, A	no
45	csa_locus_6096_iso_1_len_715_ver_2	21	7326	45.4	1	Uncharacterized protein	Trema orientale	MPGR	NME	no
46	csa_locus_3129_iso_3_len_709_ver_2	17	11165	34.2	2	Uncharacterized protein	Aquilegia coerulea	MPGR	NME, NA	no

^1^ PTMs are abbreviated as NME for N-term M excised, O for oxidation (M), NA for N-term acetylation, M for methylation (K), D for dimethylation (K), S for succinylation (K), A for acetylation (K), P for phosphorylation (STY); multiple modifications of the same type are grouped as one PTM (e.g., multiple acetylations are noted with a single A). MPGR: Medicinal Plant Genomic Resource. ^2^ Proteins identified by BUP pin [1].

## Supplementary Materials

The following are available online https://www.mdpi.com/2227-7382/7/4/33/s1, Figure S1: Relationship between the molecular weight (MW) of the protein standards analysed and their sequencing results by top-down proteomics, Figure S2: Mascot search results of protein standards MS/MS peak lists using (A) a homemade database and (B) Swissprot database, Figure S3: Distribution of cannabis proteins according to their accurate masses (3–70 kDa), Figure S4: Multivariate statistical analyses using LC-MS data from cannabis protein samples. (A) Principal Component Analysis (PCA); (B) Hierarchical Clustering Analysis (HCA), Figure S5: Statistics on parent ions from cannabis proteins analysed by LC-MS/MS. (A) Distribution of deconvoluted mass according to their charge state; (B) Distribution of deconvoluted masses according to their base peak intensity; (C) Distribution of deconvoluted masses according to their elution times, Figure S6: Top-down sequencing result from Mascot for *C. sativa* Cytochrome b559 subunit alpha (A0A0C5ARS8). (A) Protein view; (B) Peptide view, Table S1: Summary of Mascot results for standards and cannabis samples using various databases, dynamic modifications, and fragment tolerance, Table S2: List of proteins identified from standards samples using Mascot algorithm and either a homemade database or Swissprot database, Table S3: List of proteoforms identified from standards samples using Mascot algorithm and either a homemade database or Swissprot database, Table S4: List of proteins identified from medicinal cannabis protein samples using Mascot algorithm and UniProtKB Cannabis sativa or SwissProt databases, Table S5: List of proteoforms identified from protein standards samples using Mascot algorithm with 50 ppm fragment tolerance and UniProtKB C. sativa database.
